# Chemical and biochemical characterization of *Ipomoea aquatica*: genoprotective potential and inhibitory mechanism of its phytochemicals against α-amylase and α-glucosidase

**DOI:** 10.3389/fnut.2023.1304903

**Published:** 2023-12-21

**Authors:** Kangkon Saikia, Saurav Dey, Shabiha Nudrat Hazarika, Gautam Kumar Handique, Debajit Thakur, Arun Kumar Handique

**Affiliations:** ^1^Department of Biotechnology, Gauhati University, Guwahati, Assam, India; ^2^Life Sciences Division, Institute of Advanced Study in Science and Technology, Guwahati, Assam, India; ^3^Guwahati Biotech Park, Guwahati, Assam, India; ^4^Department of Botany, Nalbari College, Nalbari, Assam, India

**Keywords:** *Ipomoea aquatica*, phytochemical composition, biochemical composition, HRMS, alpha amylase inhibition, alpha glucosidase inhibition, inhibition kinetics, DNA damage prevention

## Abstract

*Ipomea aquatica,* also known as water spinach, is an aquatic non-conventional leafy vegetable and is considered a healthy and seasonal delicacy in ethnic food culture. The study revealed the presence of rich chemical and biochemical composition in *I. aquatica* and antioxidant activities. Moreover, the plant extracts demonstrated significant DNA damage prevention activity against UV/H_2_O_2_-induced oxidative damage. High-resolution mass spectrometric analysis by UPLC-qTOF-MS/MS resulted in the identification of over 65 different compounds and 36 important secondary metabolites. Most of the compounds identified represented polyphenolic compounds, *viz.* polyphenol glycosides and phenolic acids, followed by alkaloids and terpenoids. A UPLC-DAD method was developed and quantified for 10 different polyphenolic compounds. Out of all the metabolites examined, a significant number of compounds were reported to have various bioactive properties, including antibacterial, antiviral, antitumor, hepatoprotection, and anti-depressant effects. The plant extracts were found to contain various compounds, including euphornin, lucidenic acid, and myricitin glycosides, which possess significant medicinal value. Metabolite analysis utilizing GC–MS revealed the presence of various fatty acids, amino acids, sugars, and organic acids. The analysis revealed the presence of essential unsaturated fatty acids such as α-linolenic acid as well as beneficial substances such as squalene., The evaluation of glycemic control activity was carried out by comprehending the inhibitory potential of α-amylase and α-glucosidase, outlining the kinetics of the inhibition process. The inhibitory activities were compared to those of acarbose and revealed stronger inhibition of α-glucosidase as compared to α-amylase. Furthermore, the mechanism of inhibition was determined using *in silico* analysis, which involved molecular docking and molecular dynamic simulation of the identified IA phytochemicals complexed with the hydrolase enzymes. The study generates convincing evidence that dietary intake of *I. aquatica* provides a positive influence on glycemic control along with various health-protective and health-promoting benefits.

## 1 Introduction

Lifestyle diseases are emerging as an alarming global issue, particularly among urban populations. Pathologies like diabetes, fatty liver, coronary heart disease, anxiety, and depression have become a growing public health concern worldwide. Clinical control of these diseases is still not sufficiently effective, and the only pertaining solution is a preventive approach. The utilization of traditional knowledge-based approach such as dietary consumption of wild edible plants has been age-old practice for prevention of these lifestyle diseases and for promotion of health improvement (1–3). Many lesser-known, non-conventional food plants, which were earlier a part of ancient food culture are widely known for their functional benefits (4, 5). Such health protecting properties of these plants are directly attributable to the presence of important phytochemicals, micronutrients, and impressive amount of dietary antioxidants (6–8). Apart from that many non-conventional food plants are also a rich source of bioactive natural products which are nowadays classified as phytopharmaceuticals.

*Ipomoea aquatica* Forsskal (IA) also known as water spinach, is a wild edible food plant found predominantly in several regions of Asia, Africa and Australia. It is a free-floating aquatic herb with long soft stems which grow in ponds, wetlands and other aquatic habitat. It is perennial but remain dormant during winter and begin to grow with the advent of pre-monsoon (April/May) and proliferate vigorously during monsoon season upto August/September. The freshly collected leafy shoot is consumed as vegetable. Many different cultivars of IA have been reported in North East India where the plant occurs abundantly in wild and traditionally known for its great medicinal and nutritional value. The plant is also nowadays cultivated in some regions for consumption as leafy greens. Being wild or semi- wild IA is resilient to harsh climatic conditions and hence easy to cultivate as compared to other conventional leafy vegetables. IA has long been used in anti-diabetic therapy, and according to Indian Ayurveda, green leaves of IA are recommended to be consumed orally for the treatment of diabetes. IA has been recently demonstrated for antidiabetic activity in *in vivo* experiments, but its prospect as food based glycemic control is not properly established (9). Moreover, previous works demonstrated the potential of IA extract to exhibit pharmacological activities including prevention of liver injury, anticancer activities and hypoglycemic effects (3, 10, 11). In contrast, these activities are attributed directly toward its phytochemical makeup. Although, the chemical composition of few IA cultivars has been explored earlier, the values differ significantly from region to region and even across the cultivars. Likewise, the phytochemicals identified in these works are often restricted to a small number of commonly found polyphenols, and the quantitative content of these compounds in IA is yet to be determined.

The phytochemical composition of many non-conventional leafy vegetables is often reported for their significant inhibitory activity toward glycoside hydrolases like α-amylase (AML) and α-glucosidase (YGU) (12–14). AML and YGU play a key role in the conversion of dietary carbohydrates to oligosaccharides and glucose. Previous studies suggest that IA has the ability to inhibit glucose absorption in a perfused rat intestinal preparation as well as to act on AML and YGU. However, the mechanism of the AML and AGU inhibition by IA remains unexplored. In contrast, the mechanism and proportion of inhibition of AML and YGU has a significant role in the reduction of postprandial glycemia, which is potential factor for development of type 2 diabetes (12, 14). Furthermore, it is crucial to consider the dietary management of postprandial glycemia as an essential concern for patients who have already been diagnosed with type 2 diabetes. Phytochemicals, particularly polyphenols, are among the most extensively studied natural compounds that are demonstrated to act on these enzymes (13). Conventional leafy vegetables do not contain sufficient quantities of dietary polyphenols; hence, wild edible plants containing a rich phytochemical makeup are far more effective at exerting inhibitory effect on these enzymes. Furthermore, the quantitative content of the phytochemicals is another crucial factor that greatly contributes to the synergistic inhibition mechanism and kinetics of AML and YGU inhibition.

The present study was undertaken with three main objectives: first, to evaluate IA for its chemical composition, and *in vitro* antioxidant activities, including oxidative DNA damage prevention. The second objective involved the identification of the phytochemical constituents of IA using cutting-edge high resolution mass spectrometry (HRMS) techniques, along with quantification of some important polyphenolic compounds. Finally, the study aimed to evaluate the inhibitory effects of IA extract on AML and YGU, highlighting the inhibition kinetics and mechanism.

## 2 Materials and methods

### 2.1 Chemicals and reagents

All the chemicals used for analytical work were of ACS grade. Porcine pancreatic α-amylase (EC 3.2.1.1, A3176) and α-glucosidase from *Saccharomyces cerevisiae* (EC 3.2.1.20, G5003) were obtained from Sigma-Aldrich. Phosphate buffer and soluble starch were obtained from HiMedia Laboratories (India); 4-Nitrophenyl β-D-glucopyranoside (N7006) was obtained from Sigma-Aldrich. Standard polyphenolic compounds *viz.* gallic acid, chlorogenic acid, 4-hydroxybenzoic acid, vanillic acid, caffeic acid, rutin, sinapic acid, ferulic acid, naringin, and quercetin were of HPLC reference standard grade and obtained from Sigma-Aldrich.

### 2.2 Collection of plant material

Edible parts of *Ipomoea aquatica* (IA) consisting of leaves and tender shoots were obtained from wild natural habitats such as wetlands of the state of Assam, India in the month of July. Freshly collected leaves were washed, cleaned and freeze dried. The moisture content of the leaves was calculated according to the method outlined by AOAC method (930.15). The dry leaves were then ground to fine powder resulting ground dry matter (dm) and used for subsequent analysis.

### 2.3 Determination of proximate composition and amino acid content

The proximate composition and mineral profile in IA were determined on dry weight basis using respective AOAC methods (15) as mentioned in Supplementary Table S1. For estimation of total carbohydrate, the dry matter of the leaves were initially digested by 2.5 N HCl and estimated by anthrone reagent (16). The calorific value (kcal/100 g) was calculated from the given formula (17).


$$C=\left(4\times C\right)+\left(4\times P\right)+\left(9\times L\right)$$

where, C, P and L are percentage of carbohydrate, protein and lipid content, respectively.

Extraction of amino acids was carried out according to method 994.12 of AOAC (15) with some modifications (18). Extracted amino acids were derivatized using FMOC and OPA followed by quantification in HPLC-DAD (Agilent, United States). The detailed method of amino acid extraction, derivatization and quantification is listed in Supplementary material.

### 2.4 Polyphenols content and *in vitro* antioxidant activity

The phytochemicals of IA were extracted using a hydroalcoholic solvent system consisting of 80% methanol. This solvent system has been previously demonstrated to effectively extract various phenolic components, including flavonoids and other secondary metabolites (19). 5 g of the ground dry matter of IA was extracted under constant agitation for 6 h at room temperature using about 100 mL of 80% methanol. The components were then sonicated for 30 min and extracted again for 90 min in water bath shaker at 40°C. The extracts were centrifuged, and concentrated using a rotary evaporator at 35°C and the remaining content was lyophilized. A portion of the lyophilized extract was reconstituted in HPLC grade methanol for subsequent phytochemical analysis.

Total phenolics content (TPC) was estimated by Folin–Ciocalteu method (20) and the results were expressed as gallic acid equivalent (mg GAE/g dm). Total flavonoid content (TFC) was estimated by aluminum chloride method (21) and expressed as rutin equivalent (mg RE/g dm).

*In vitro* antioxidant activity of the reconstituted crude IA extracts was determined using five different assay methods namely DPPH radical scavenging assay (22), Trolox equivalent antioxidant capacity (TEAC) assay (23), oxygen radical absorbance capacity (ORAC) assay (24), ferric reducing antioxidant power (FRAP) assay (25) and phosphomolybdate assay for total antioxidant capacity (TAC) (26).

### 2.5 UPLC-ESI-QTOF-MS based phytochemical identification

The screening of different phytochemicals from the crude extract was carried out using an Agilent, 6,550 iFunnel quadrupole time of flight (QTOF) mass coupled with and Agilent LC/MS system. The LC method was optimized to separate and detect the maximum possible phytoconstituents from IA crude extract. 5 μL of redissolved lyophilized extract was injected using an autosampler into a reverse-phased column thermostatically maintained at 40°C. The mobile phase comprised of (A) 0.1% formic acid in water; (B) 90% acetonitrile with 10% water and 0.1% formic acid. The flow rate was 0.3 mL/min, and the gradient elution program was set as 95%A for 1 min 0%A for 20 min and 25 min 95%A for 26 min and 30 min.

Mass spectrometric acquisition was done in both positive and negative ESI mode using dual AJS ESI within the range of 100 to 1,100 m/z with MS and MS/MS scan rate 1 spectra/s. The scan source parameters were: VCap 3,500, nozzle voltage 1,000 V, fragmentor 175, skimmer 65 and octupole RFPeak 750. The temperature of gas and sheath gas were 250°C and 300°C, respectively, with flow of 13 L/min and 11 L/min. The data acquisition was performed by Mass Hunter software of Agilent and the detected compounds were identified by searching against a plant metabolite database available with the instrument.

### 2.6 Quantitative profiling of polyphenols using UPLC-DAD

Among all the identified metabolites, 10 polyphenolic compounds *viz.* gallic acid, chlorogenic acid, 4-hydroxybenzoic acid, vanillic acid, caffeic acid, rutin, sinapic acid, ferulic acid, naringin, and quercetin were quantified from IA extract. Quantification was carried out in a UPLC-DAD system (Agilent, United States) with a polar RP C18 column (250 mm L, 4.6 mm ID, 5 μm particle size) using mobile phase 0.1% formic acid in HPLC grade water (A) and 100% acetonitrile (B). The column was thermostatically maintained at 40°C, flow rate was set at 1 mL/min and 15 μL was used as injection volume. The gradient program for separation was as follows: 90%B for 0 min, 70%B for 8 min, 50%B for 13 min, 40%B for 15 min, 90%B for 18 min and 20 min. DAD detector was set at 255 nm for 4-hydroxybenzoic acid, chlorogenic acid, and vanillic acid; 280 nm for cinnamic acid, gallic acid, naringin and p-coumaric acid; 320 nm for caffeic acid, sinapic acid and ferulic acid; 375 nm for rutin and quercetin.

### 2.7 GC–MS analysis

The metabolite composition of IA was determined by GC 2010 plus with TP-8030 triple quadrupole MS (Shimadzu, Japan), fitted with EB-5 MS column (30 m); briefly – a portion of lyophilized IA extract was mixed with 200 μL methoximation mixture and incubated at 37°C for 90 min. This was followed by the addition of 150 μL BSTFA with 1% trimethylchlorosilane and incubation at 70°C for 60 min. 1 μL of the test portion was injected in splitless mode. Helium was used as carrier gas at a flow of 1 mL/min; columns were isothermally maintained at 80°C for 2 min followed by ramping at 10°C/min to 300°C followed by a hold for 6 min. Injector and ion source temperatures were kept at 280°C and 230°C along with 150°C transfer temperature to MS. The ionized mass fragments were recorded between 50–550 m/z.

### 2.8 Oxidative DNA damage prevention assay

Qualitative assessment of oxidative DNA damage prevention (genoprotective effect) of IA extract was carried out on PTZ57R (2,886 bp) plasmid (Fermentus, United States). Lyophilized IA extract was reconstituted on HPLC grade water. The reaction mixture was prepared by adding 16 μL TE buffer with 2 μL DNA and 5 μL extract at different concentrations (1–10 μg/mL) and 2 μL 30% H_2_O_2_. The negative control comprised of 5 μL water; a reaction as control was set without the addition of H_2_O_2_. The reaction mixture was exposed to UVC radiation for 15 min to produce hydroxyl radicals and induce DNA damage. After that, the reaction was terminated by the addition of loading dye (6X). The mixtures were transferred to a 1% agarose gel and electrophoresis was carried out followed by documentation in a gel documentation system. The densitometric processing of the gel image was carried out in ImageJ tool (27). The percentage of DNA damage inhibition (DI) was calculated by the following equation.


$$DI=\frac{1-N_{s}}{N_{0}}\times 100$$

where, *N_S_* and *N_0_* are percentage of nicked DNA in lanes with sample and without sample extracts, respectively.

### 2.9 α-amylase and α-glucosidase inhibitory assays

For determination of inhibitory effects of IA on α-amylase, 0.5 U/mL solution porcine pancreatic α-amylase (PPA) was prepared in 0.2 M phosphate buffer, pH 6.5. Subsequently, 0.5 mL of PPA was incubated with different concentrations of 1 mL IA extracts (prepared in phosphate buffer) at 37°C for 30 min. This reaction was initiated by the addition of 0.5 mL soluble starch solution (5 mg/mL) and allowed to be hydrolyzed for 15 min at the same condition. A negative control and a reaction blank were processed in a similar manner without the addition of enzyme and extract, respectively. The reaction was then stopped by adding 0.3 mL dinitro salicylic acid (DNS) reagent and diluted using 0.5 mL phosphate buffer. The reaction mixture was then heated in a boiling water bath for 5 min and allowed to cool at room temperature. The absorbance of the colored complex was recorded at 540 nm using Varioskan™ LUX multimode microplate reader (Thermo Scientific, United States).

For α-glucosidase inhibitory assay, 0.15 U/mL α-glucosidase from *Saccharomyces cerevisiae* (YGU) was prepared in 0.2 M phosphate buffer, pH 6.5. The p-nitrophenyl α-D-glucopyranoside (pNPG) was used as a reaction substrate. The reaction volume comprised of 50 μL of YGU and 150 μL of inhibitor, i.e., IA extracts at different concentrations, was incubated for 10 min at 37°C. The reaction was initiated by addition of 50 μL of 0.2 mg/mL pNPG followed by incubation for 15 min at 37°C. The reaction was then terminated by addition of 50 μL 0.2 M Na_2_CO_3_ and the absorbance of the colored solution was recorded at 450 nm using Varioskan™ LUX multimode microplate reader (Thermo Scientific, United States). The results were quantified as maltose equivalent using a calibration curve.

All the samples were measured in triplicates. The percentage of inhibition (I) for both PPA and YGU were calculated using the following formula:


$$I=\frac{\left(A_{C}-A_{CB}\right)-\left(A_{S}-A_{SB}\right)}{A_{C}-A_{CB}}\times 100$$

where, *A_C_*, *A_S,_ A_CB,_ A_SB_* are the absorbances for control, sample, control blank and sample blank, respectively.

The IC50 values for α-amylase and α-glucosidase inhibition were calculated using the following equation (28).


(1) $$I=I_{\text{max}}\left(1-\frac{IC_{50}}{\left[I\right]+IC_{50}}\right)$$

where, [I] is the concentration of IA extracts or acarbose, and *Imax* is the maximum percentage inhibition.

### 2.10 α-amylase and α-glucosidase inhibition kinetics

The kinetics of PPA inhibition were determined at different concentrations of starch in the range of 0.25–5 mg/mL. Similarly, for YGU, inhibition kinetics were determined at different concentrations of pNPG in the range of 0.1–2.0 mM. IA extract at various concentrations were used inhibitor (*[I]*); for PPA inhibitor concentration was in the range of 1–2.5 mg/mL and for YGU the range was within 0.05–0.2 mg/mL. Acarbose was used as a standard inhibitor and kinetic parameters were calculated in similar manner to that of IA extract. The enzymatic reactions and substrate utilization were measured spectrophotometrically using the same methods as described above. The reaction velocities were determined by recording the starch digestion at 0, 5, 10 and 15 min, respectively. The kinetic parameters were determined using Michaelis–Menten kinetic model where *V* is the rate of reaction, *V_max_* is the maximum reaction rate of enzyme, [S] is the substrate concentration, and *K_m_* is the Michaelis–Menten constant. The initial rate of enzyme reaction (*V_0_*) was determined from the quantity of substrate hydrolyzed at different concentrations by plotting a calibration curve. The nature of inhibition was determined by plotting a Lineweaver-Burk double reciprocal plot of *1/V* against *1/S* for each substrate concentration and IA extract concentration used as inhibitor. The Dixon plot [Eq. (2)] and Cornish Bowden-Eisenthal plot [Eq. (3)] was used to calculate the binding constant *K_i_*, the equation used was as follows:


(2) $$V=\frac{V_{\text{max}}\left[S\right]}{Km\left(1+\frac{\left[I\right]}{K_{i}+\left[S\right]}\right)}$$

(3) $$V=\frac{V_{\text{max}}\;\left[S\right]}{Km\;\left(1+\frac{\left[I\right]}{K_{i}}\right)+\left[S\right]\left(1+\frac{\left[I\right]}{K_{i}}\right)}$$

### 2.11 Molecular docking and molecular dynamic simulation

In order to evaluate the interaction between IA polyphenols with human α-amylase and α-glucosidase, molecular docking was carried out in the active site of both the enzymes. Three dimensional structures of human α-amylase (1HNY), porcine pancreatic α-amylase (1OSE) and α-glucosidase (3L4Y) were obtained from protein data bank (PDB). For both α-amylase and α-glucosidase structures, acarbose interaction was modeled in all the proteins from acarbose co-crystallized templates available at PDB and active sites were mapped. The proteins were processed using the protein preparation wizard of Schrödinger maestro (Schrödinger suite 2021) and docking grid was generated. Structures of all the identified compounds from IA were obtained and processed using LigPrep tool allowing generation of tautomer. All the ligands were then docked in the acarbose binding site grid of all the prepared structures. The stability of the docked complexes was determined by molecular dynamic (MD) simulations. Briefly, the system was generated using orthorhombic boundary conditions containing TIP3P water model and 0.15 M NaCl within 10 Å buffer region. The system was equilibrated at 300 K and 1 bar using NVT and NPT ensembles. Nosé-Hoover chain thermostat and Martyna Tobias Klein barostat were used with isotropic coupling at 1 ps and 2 ps relaxation times. The system was relaxed using default options and MD simulations were performed for 100 ns in Desmond (academic version 2023) using the OPLS4 force field. The binding free energy of the complexes was calculated using the MM-GBSA method.

### 2.12 Statistical analysis

All the statistical analyzes and curve fitting were carried out using R programming and SPSS Statistics v28.0 (IBM Corp, United States). Data generated were analyzed in triplicates and expressed as mean ± standard error of mean (SEM).

## 3 Results

### 3.1 Proximate composition and amino acid profile

The proximate composition of IA is listed in Table 1. The content of 8 individual mineral elements were shown in Table 2. IA exhibited relatively low protein content, analysis of amino acid profile revealed 20 amino acids with varying amounts (Table 2) and of the total amino acids 60.4% were essential amino acids. Among the essential amino acid lysine was highest with 2.141 g/100 g followed by phenylalanine with 1.891/100 g and isoleucine 1.674 g/100 g. Among the sulfur containing amino acids methionine occurred in relatively high amount which is 1.223 g/100 g and cystine in relatively low amount (0.291 g/100). Furthermore, among the aromatic amino acids, phenylalanine occurred in highest quantities (1.891 g/100 g) followed by tyrosine and tryptophan (Table 2).

**Table 1:** Major nutritional parameters of *I. aquatica* along with moisture content and calorific value.

Composition	Content
Moisture (%)	86.27 ± 1.221
Protein (%)	19.55 ± 0.107
Carbohydrate (%)	17.40 ± 0.250
Lipid (%)	2.43 ± 0.130
Crude Fiber (%)	14.58 ± 0.180
Minerals (%)	14.99 ± 0.136
Calorific Value (kcal/100 g dm)	165.7 ± 1.93
Sodium (mg/kg dm)	235.78 ± 0.999
Potassium (mg/kg dm)	1294.08 ± 0.805
Calcium (mg/kg dm)	254.15 ± 0.738
Magnesium (mg/kg dm)	196.27 ± 0.478
Manganese (mg/kg dm)	9.63 ± 0.113
Iron (mg/kg dm)	146.71 ± 0.798
Zinc (mg/kg dm)	8.11 ± 0.166
Phosphorus (mg/kg dm)	1023.12 ± 0.874

The values were reported based on dry weight (dm) and the percent composition is equivalent to g/100 g dm.

**Table 2:** Amino acid profile of *I. aquatica* (mean ± SEM).

Amino acid	g/100 g	% Total
Essential amino acids		
MET	1.223	22.83
TRP	0.459	6.26
PHE	1.891	31.89
ILE	1.674	35.54
LEU	0.917	19.46
LYS	2.141	33.95
HIS	0.952	12.65
THR	0.550	12.86
VAL	1.030	24.49
Non-essential amino acids		
ASP	0.178	1.65
GLU	1.623	32.62
SER	1.281	33.95
GLN	0.538	10.26
GLY	0.752	27.89
ARG	0.505	6.68
ALA	0.183	5.71
TYR	0.503	7.74
CYS	0.291	6.68
PRO	1.261	30.53
Secondary amino acids		
Hydroxyproline	0.142	3.02
Total	17.95	
EAA	10.836	60.4
NEAA	7.257	40.4
EAA/NEAA	1.49	

EAA, total essential amino acids; NEAA, total non-essential amino acids.

### 3.2 Polyphenols content and *in vitro* antioxidant activity

IA exhibited total phenolics and flavonoid content of 142.43 mg GAE/g dm and 43.06 mg RE/g, respectively (Table 3). Assessment of different radical scavenging capabilities revealed that IA antioxidant can effectively inhibit ABTS radicals (IC50 of 0.087 mg/mL and 0.924 mg dm eq) as compared to DPPH radicals (IC50 of 0.122 mg/mL and 1.125 mg dm eq). Different antioxidant assay activities exhibited by IA were compared to that of trolox, which is a vitamin E analog, and hence results were expressed as trolox equivalent. In both the cases radical scavenging assays the IC50 values were similar to of the standard compound trolox (Table 3). ORAC assay demonstrated significant capability of IA extract to inhibit free radical damage by measuring the fluorescence intensity. Furthermore, upon assessment of radical scavenging capability by TEAC assay, the antioxidant capacity was found to be 699.35 μmol TE/g dm. Moreover, the ferric reducing power as determined by FRAP assay was observed to be 5762.88 μmol TE/g dm. Determination of total antioxidant capacity by phosphomolybdate assay revealed antioxidant activity of 17.93 mM TE/g dm.

**Table 3:** Dietary antioxidant and *in vitro* antioxidant activity of IA.

Dietary antioxidants	
TPC (mg GAE/g dm)	42.43 ± 0.448
TFC (mg RE/g dm)	36.06 ± 0.913
*In vitro* antioxidant activity	
DPPH IC50 (mg/mL)	0.122
DPPH IC50 of Trolox (mg/mL)	0.073
ABTS IC50 (mg/mL)	0.087
ABTS IC50 of Trolox (mg/mL)	0.096
TEAC μmol TE/g dm	699.35 ± 0.959
ORAC μmol TE/g dm	968.23 ± 0.876
FRAP μmol TE/g dm	5762.88 ± 1.552
TAC (mM TE/g dm)	17.93 ± 0.257

### 3.3 LC-ESI-QTOF-MS based phytochemical screening

**Figure 1:**
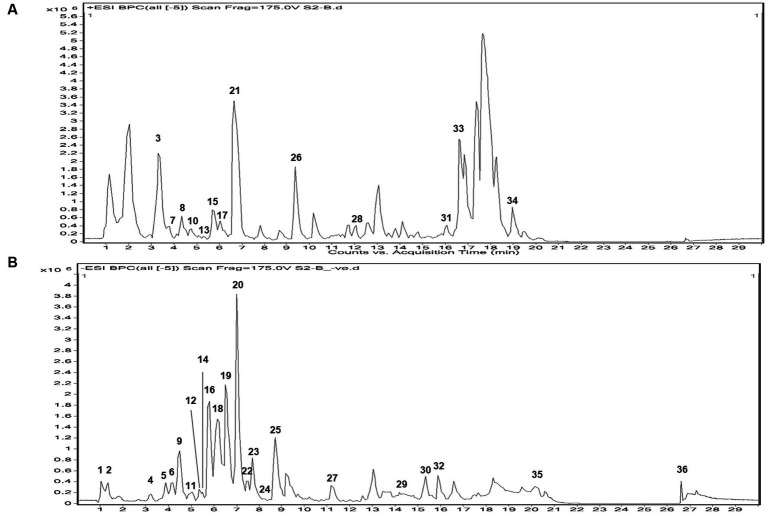
Total ion chromatogram of UPLC-ESI-qTOF-MS analysis in **(A)** + ESI mode and **(B)** – ESI mode, of IA extract.

In both positive and negative ESI mode, LC-QTOF-MS analysis has identified more than 65 compounds, and among them 36 compounds were important secondary metabolites (Tables 4, 5). The compounds were identified by database search of HRMS data and similarity percentage of the retrieved compounds were reported. Moreover, characteristic mass fragments of the compound hits were also listed. Total ion chromatograms in the ionization mode were shown in Figure 1. Among the compounds identified, the number of polyphenols and their derivatives were high, followed by some important alkaloids and terpenoids. Polyphenolic compounds such as phenolic acids and flavonoids were detected mostly in negative ESI mode whereas alkaloids and terpenoids were detected mostly in positive ESI mode. Major polyphenolic compounds in IA were comprised of quinic acid, caffeoylquinic acid and its derivatives including caffeic acid and chlorogenic acid. Furthermore, flavone glycosides constituted a dominant portion of IA phytochemical constituents. In addition, non-polyphenolic compounds such as norharman, asclepin, jervine, edulinine, cerbertine, euphornine, jatrophone etc. were also found to be a part of IA phytochemical constituent, which had not been reported in earlier works. Apart from the 36 secondary metabolites identified, the other compounds such as fatty acids, amino acid derivatives etc. which were obtained in database search are listed separately in Supplementary Tables S3, S4.

**Table 4:** LC-ESI-qTOF-MS/MS determination of phytochemical composition of IA crude extract in positive ionization (+ESI) mode.

	RT (min)	Tentative compound name	Ionization model	Base peak	Fragment ions	Similarity (%)
1	3.08	Larixinic Acid	[M + H]+	127.0387	127, 43, 53, 109	95.68
2	4.09	Feruloyl-2-hydroxyputrescine	[M + Na]+	195.0894	85, 86, 149, 103	79.88
3	4.89	3-Hydroxycoumarin	[M + H]+	163.0368	117, 119, 107, 77	93.89
4	4.84	Norharman (β-Carboline)	[M + H]+	169.0751	169, 115, 168, 142	79.25
5	5.54	Solanocapsine	[M + Na]+	100.1109	123, 95, 166, 93, 180, 81	76.20
6	5.86	Quercetin	[M + H]+	303.0481	153, 303, 69, 139, 257	92.73
7	6.13	Hesperetin 7-O-glucuronide	[M + Na]+	163.038	147,151,125,137,177,291	98.96
8	7.14	(S)-Edulinine	[M + Na]+	177.0537	188, 106, 174, 202, 79, 134	95.01
9	9.39	3-tert-Butyl-5-methylpyrocatechol	[M + H]+	181.1212	57, 65, 67, 121, 165	93.56
10	12.06	Cerbertin	[M + Na]+	567.2552	357, 45, 263, 161, 605	97.63
11	16.08	Asclepin	[M + Na]+	503.2405	371, 373, 383, 313, 429	96.91
12	16.83	Camptothecin	[M + H]+	362.8563	247, 235, 249, 245, 347	96.48
13	19.17	Euphornin	[M + H]+	547.2683	401, 417, 421, 463, 525	97.48
14	20.33	Methyl 2-(10-heptadecenyl)-6-hydroxybenzoate	[M + Na]+	389.305	345, 331, 221, 163	99.74

**Table 5:** LC-ESI-qTOF-MS/MS determination of phytochemical composition of IA crude extract in negative ionization (-ESI) mode.

	RT (min)	Tentative compound name	Ionization model	Base Peak	Fragment ions	Similarity (%)
1	1.08	Quinic acid	[M-H]-	191.0552	85, 93, 59, 45	93.42
2	1.70	Gallic acid	[M-H]-	169.0137	125, 169, 81, 97	78.21
3	3.08	1-O-Caffeoylquinic acid	[M-H]-	191.0555	173, 179, 85, 353, 191	94.80
4	3.86	4-Hydroxybenzoic acid	[M-H]-	138.1185	93, 137, 94	89.25
5	4.00	Cis-5-Caffeoylquinic acid	[M-H]-	191.055	191, 179, 135, 352	93.52
6	4.43	Caffeic acid	[M-H]-	135.0433	134, 89, 135, 132, 79, 65	92.98
7	5.08	Herbacetin 3,8-diglucoside	[M-H]-	300.0255	179, 299, 301, 343, 43	95.81
8	5.51	Vanillic acid	[M-H]-	167.0344	151, 123, 107, 95, 83, 65	90.36
9	5.77	Myricetin 7-rhamnoside	[M-H]-	300.0262	317, 165, 247, 275, 135	97.49
10	5.84	Oolonghomobisflavan A	[M-H]-	928.1698	169, 319, 125, 151, 261	75.53
11	6.38	Chlorogenic acid	[M-H]-	335.0765	190,135,180	76.41
12	6.69	1,4-Di-O-caffeoylquinic acid	[M-H]-	179.0351	177,161,307,309,133	98.18
13	7.06	6”-Caffeoylhyperin	[M-H]-	300.026	151,137,65,579,434,137	99.77
14	7.47	Kaempferol 3-(2″,3″-diacetyl-4″-p-coumaroylrhamnoside)	[M-H]-	191.0553	59, 285, 125, 163, 145, 41	99.78
15	7.68	Rutin	[M-H]-	457.1354	298, 607, 272	87.63
16	8.18	Quercetin-3β-D-glucoside	[M-H]-	301.0378	299,135,43,109,283,59	86.14
17	8.91	Ferulic acid	[M-H]-	193.0501	89,134,133,97,59	75.23
18	11.23	Sinapic acid	[M-H]-	223.0105	161,177,205,105,45,55	83.24
19	14.65	Lucidenic acid F	[M-H]-	134.0378	59,41,439	99.46
20	15.29	Ferulic acid 4-O-glucuronide	[M-H]-	369.261	367,149,59,221,147,193	78.29
21	16.19	Jervine	[M-H]-	164.0714	422,424,297,299,390,94	80.99
22	26.72	Jatrophone	[M-H]-	311.1666	295, 269, 241, 285	87.17

### 3.4 Quantitative determination of polyphenols

**Figure 2:**
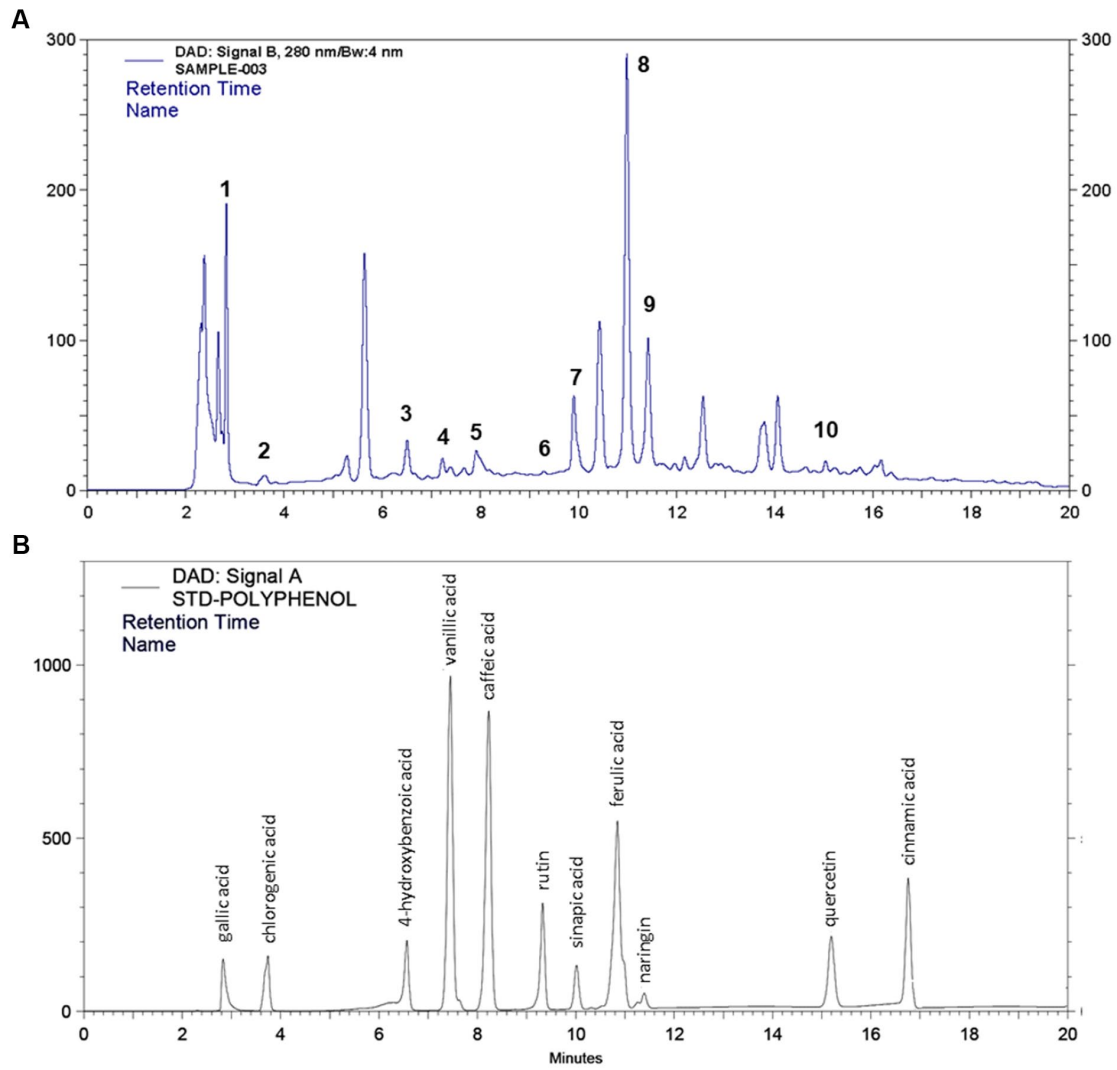
HPLC quantification of 11 different polyphenol compounds from **(A)** IA crude extract and **(B)** standard polyphenol compounds.

After identification of different polyphenols in LC-ESI-QTOF-MS analysis 10 major polyphenolic compounds were further quantified using a customized UPLC-DAD method (Figure 2). The quantitative composition of the individual polyphenolic compound is listed in Table 6 and the parameters of the quantification method are listed in Supplementary Table S6. Among the quantified polyphenols prominent were caffeic acid (1553.8 μg/g) and ferulic acid (222.1 μg/g). Furthermore, contents of quercetin and chlorogenic acids were also found marginally high, which was 53.22 μg/g and 27.15 μg/g, respectively. Gallic acid, 4-hydroxybenzoic acid and naringin content were found to be low and varied in the range of 10–15 μg/g.

**Table 6:** List of polyphenols quantified from IA extracts using UPLC-DAD.

	Compound name	RT (min)	Concentration (μg/g)
1	Gallic acid	2.83	10.42 ± 0.636
2	Chlorogenic acid	3.61	27.15 ± 0.494
3	4-hydroxy benzoic acid	6.51	29.09 ± 0.737
4	Vanillic acid	7.39	17.91 ± 0.502
5	Caffeic acid	7.92	1553.8 ± 6.840
6	Rutin	9.3	32.68 ± 0.815
7	sinapic acid	9.91	24.9 ± 1.691
8	Ferulic acid	10.98	222.1 ± 1.403
9	Naringin	10.99	15.18 ± 0.396
10	Quercetin	14.81	53.22 ± 0.887

### 3.5 GC/MS analysis

GC–MS evaluation of metabolites revealed 65 metabolites which were mainly organic acids, fatty acids, amino acids, sugars, and other metabolites such as catecholamines, sterols etc. The compounds were listed based on their category, retention time and percent area of the peak (Table 7). The majority of compounds detected were organic acids, sugar and their derivatives, followed by fatty acids. The analysis revealed the presence of essential fatty acids, mostly poly unsaturated fatty acids along with omega 3 fatty acids such as α-linolenic acid and omega 6 fatty acid such as linoleic which are very rare in conventional food plants. Moreover, the presence of α-linolenic acid was also observed during UPLC-qTOF-MS analysis (as listed in Supplementary Tables S3, S4). Four essential amino acids *viz.* valine, isoleucine, threonine and tryptophan were detected in metabolic analysis as well as during HPLC quantification of amino acids. Furthermore, the present analysis also identified other plant secondary metabolites such as linalool, squalene etc.

**Table 7:** Metabolic profile of derivatized IA extract determined by GC/MS analysis.

Compound name	RT (min)	Base m/z	Area (%)
Organic acids			
L-(+)-Lactic acid	5.48	116.95	4.65
2-Propenoic acid	5.85	147.05	1.17
Oxalic acid	6.72	147.05	1.52
Butanoic acid	6.99	147.05	1.18
Benzeneacetic acid	9.13	73.05	0.52
Butanedioic acid	9.30	147.05	0.58
Propanoic acid	9.53	73	1.14
Pentanedioic acid	10.58	147.05	0.32
Dihydroxybutanoic acid	10.95	73	0.88
Malic acid	11.70	147.05	2.18
Benzoic acid	12.75	73.05	0.26
Adipic acid	17.76	157.1	0.12
Phthalic acid	22.57	149.05	0.06
Fumaric acid	24.09	221.1	0.08
Fatty acids			
9-Decenoic acid	11.26	73.05	0.38
Eicosanoic acid	15.03	73.05	0.06
1-Nonadecene	15.49	83.1	0.16
cis-4-Decenedioic acid	16.09	73.05	0.21
n-Hexadecanoic acid	17.40	73	1.93
Nonadecene	17.62	97.1	0.33
Dodecanoic acid	17.81	73.05	0.03
Linoleic acid	19.05	67.05	0.35
Alpha linolenic acid	19.15	79.05	0.31
Octadecanoic acid	19.35	73.05	1.33
Docosanoic acid	22.80	73.05	0.05
Amino acid and derivatives			
L-Valine	5.95	72.05	1.49
l-Isoleucine	7.31	86.1	0.35
Serine	8.50	116.05	2.03
l-Threonine	9.07	117.05	1.64
Glycine	9.23	174.1	0.36
L-Homoserine	9.97	146.1	0.29
Aspartic acid	10.89	116.15	3.33
Pyroglutamic acid	12.27	84	2.27
L-Tryptophan	16.63	202.15	0.17
Sugar and derivatives			
D-(−)-Erythrose	11.04	73.05	0.49
Pentitol	12.37	73.05	1.84
L-Fucitol	12.59	219.15	2.32
2-Deoxy-D-ribose	12.81	73.05	0.28
D-Arabinopyranose	13.00	73.05	0.15
D-Erythro-Pentitol	13.14	103.05	0.4
D-(−)-Ribofuranose	13.27	217.15	0.16
Xylonic acid	13.58	73.05	0.97
L-Gluconic acid	13.78	73.05	0.78
D-Fructose	13.90	217.15	1.75
D-(−)-Lyxose	14.09	217.25	1.99
Xylitol	14.60	218.05	2.98
D-(−)-Rhamnose	14.69	117.05	0.11
D-Allose	16.51	73.05	0.58
D-(+)-Talose	16.57	73.05	0.68
glucitol	17.00	73.05	0.24
D-Galactofuranose	17.57	217.15	0.21
Dulcitol	24.28	73.05	0.13
Other			
Tetraethylene glycol	7.66	147.9	2.33
Octopamine	8.65	174.1	0.63
Diglycerol	8.71	147.85	3.08
L-Threitol	11.86	73.05	0.76
2-Mono-isobutyrin	11.91	73.05	0.59
Erythritol	11.98	217.25	2.17
Myo Inositol	18.62	73.05	4.25
Linalool	18.71	131.1	0.15
1-Heptacosanol	19.55	97.1	0.34
Phytol acetate	19.73	68.05	0.22
Squalene	24.71	69.05	0.11
Ethanethioic acid	25.15	281.05	0.02

### 3.6 Oxidative DNA damage prevention

**Figure 3:**
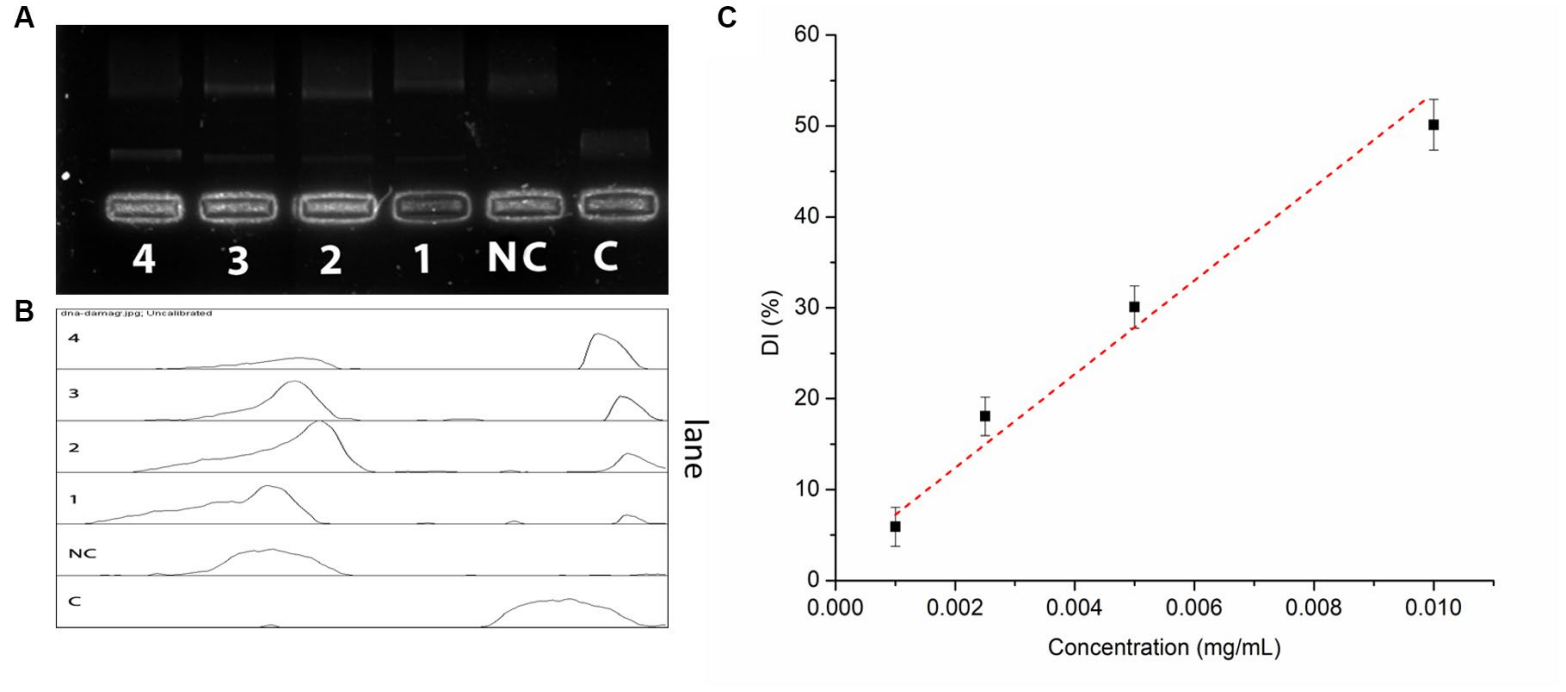
Oxidative DNA damage prevention assay by IA extract **(A)** electrophoretic separation of nicked DNA at different concentrations, C – control and NC – negative control; **(B)** pixel densitometry plot and **(C)** calibration curve of DNA damage prevention at different concentrations of IA extract.

IA crude extracts have exhibited a promising DNA damage prevention activity. The hydroxyl radicals produced during the UVC irradiation have conferred to a total 93.5% of DNA damage. However, the presence of IA extract has inhibited 48.9% of DNA damage at concentration of 10 μg/mL and 18.8% of DNA damage at lowest concentration levels up to 1 μg/mL (Figure 3). The DNA damage inhibition by different concentrations of IA crude extracts were represented by a calibration curve shown in (Figure 3C).

### 3.7 α-Amylase and α-glucosidase inhibition

**Figure 4:**
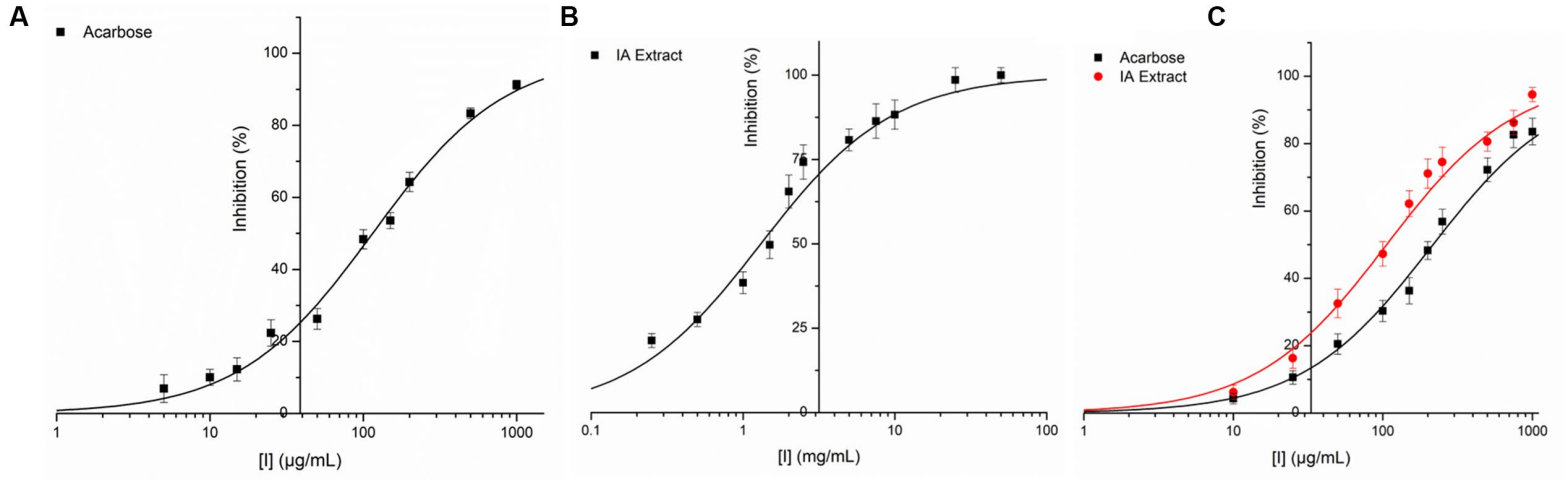
Inhibition of PPA and YGL by IA extract and acarbose at different concentrations. **(A)** PPA inhibition by acarbose, **(B)** PPA inhibition by IA extract and **(C)** YGL inhibition by both acarbose and IA extract. Curve fitting was carried out based on equation 2 with natural logarithm of X axis values.

IA crude extracts have exhibited significant levels of inhibition for both the hydrolase enzymes as represented by IC50 values (Table 8). However, YGU inhibition potential was substantially stronger as compared to PPA. The IC50 values of IA inhibition were 0.106 mg/mL for YGU and 1.316 mg/mL for PPA. Likewise, for acarbose IC50 values were 0.210 mg/mL for YGU and 0.115 mg/mL for PPA. The inhibition potential of IA extract was compared to acarbose (Figure 4) and observed that IA extracts have a significantly higher YGU inhibition potential as compared to acarbose. However, in the case of PPA inhibition acarbose inhibition was marginally high.

**Table 8:** α-amylase and α-glucosidase inhibitory activities and inhibition kinetic parameters of IA extract.

Enzyme	IC50 mg/mL	IA extract concentration (mg/mL)	V_max_ (mM/min)	K_m_ (mM)	Nature of inhibition	K_i_ (μg/mL)
α-amylase	1.316	0	0.364	0.815	Mixed	278.5
		1.0	0.296	1.429		
		1.5	0.244	1.590		
		2.0	0.165	1.628		
		2.5	0.162	2.165		
α-glucosidase	0.106	0	0.029	0.273	Mixed	17.44
		0.05	0.024	0.574		
		0.10	0.020	0.698		
		0.15	0.016	0.835		
		0.20	0.011	1.027		

### 3.8 Kinetics of α-amylase and α-glucosidase inhibition

**Figure 5:**
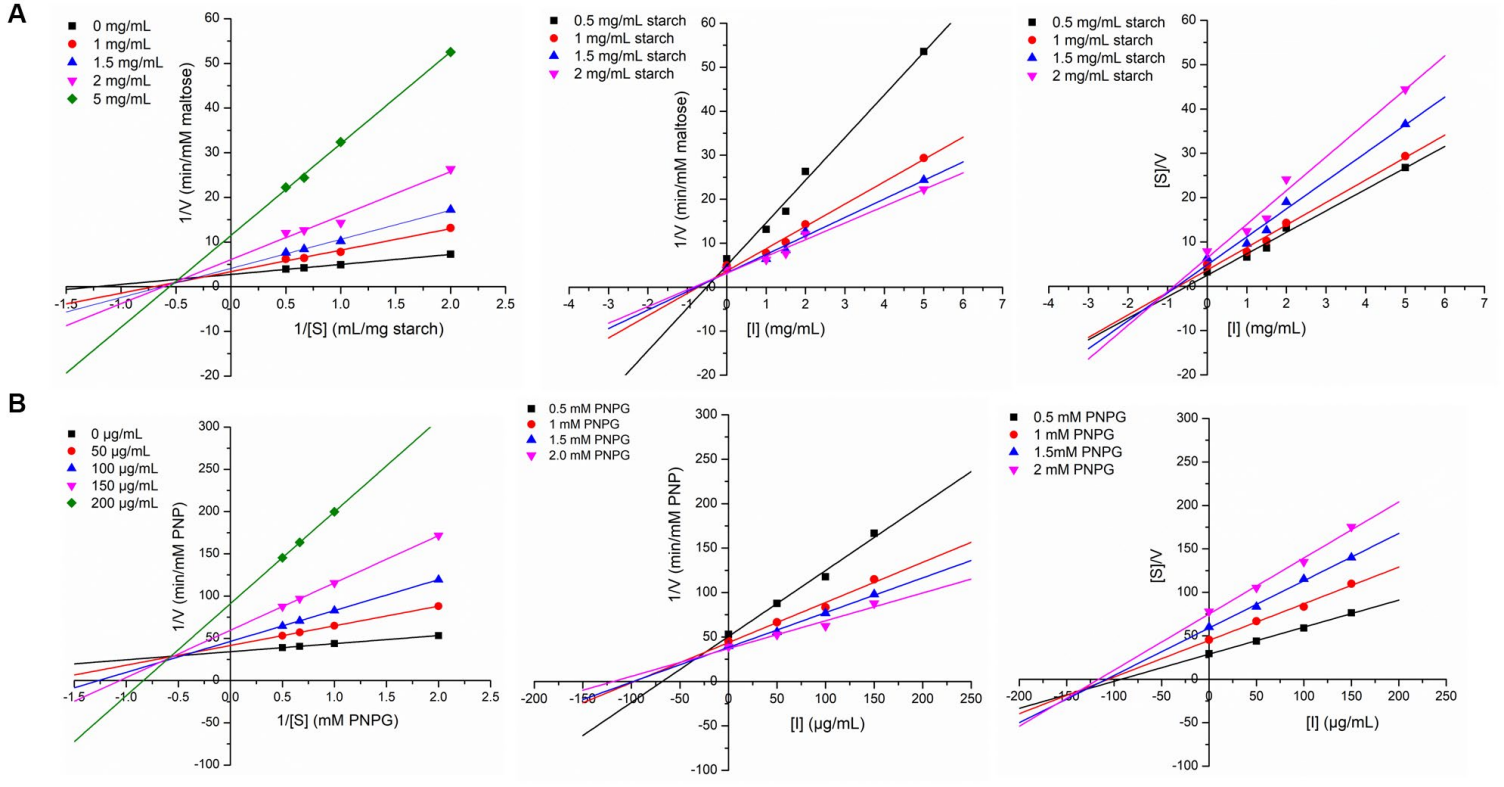
Linewaver-Burk plot, Dixon plot and Cornsih-Bowden -Esenthal plot (left to right) for IA extract inhibition at different concentration of substrate and inhibitor against **(A)** porcine pancreatic α-amylase (PPA), **(B)** yeast α-glucosidase (YGL).

In order to explore the inhibition kinetics of IA phytochemicals against PPA and YGU, the enzyme kinetic reactions of both the enzymes were investigated with different concentrations of their substrate and IA extract as inhibitor. The kinetics of inhibition was compared to that of acarbose. In order to outline the inhibitory mechanism, the Lineweaver-Burk plots (1/[S] against 1/V) were generated as shown in Figure 5. IA extracts at different concentrations were taken as inhibitors and represented by a series of lines with different slopes and intercept at Y axis. With increasing concentration of IA extracts the value of slope (*Km*/*Vmax*) as well as the intercepts (1/*Vmax*) has increased significantly. This indicated the decrease of *Vmax* with increase of *Km* which is a nature of mixed inhibition type. The same nature of inhibition toward PPA was also observed in case of acarbose, however, in case of YGU the nature of inhibition exhibited by acarbose was different. In case of acarbose YGU inhibition, *Vmax* remained constant with increase of *Km* representing a competitive inhibition, unlike IA extract which was mixed. All the kinetic parameters of PPA and YGU inhibition were listed in Table 8. Dixon plot was generated by plotting concentration of inhibitor (*[I]*) against *Km/Vmax* values (Figure 5). By comparison of *Ki* values, it was observed that IA exhibited *Ki* value of 0.278 mg/mL for PPA and 0.017 mg/mL for YGU. Moreover, smaller Ki value YGU inhibition as compared to PPA inhibition indicates that the extracts can strongly inhibit YGU as compared to PPA. Similarly, upon comparison with *Ki* of acarbose – YGU inhibition (43.12 μg/mL) it has been revealed that IA has potentially high inhibitory effects toward YGU.

### 3.9 Molecular docking and molecular dynamic simulation

**Figure 6:**
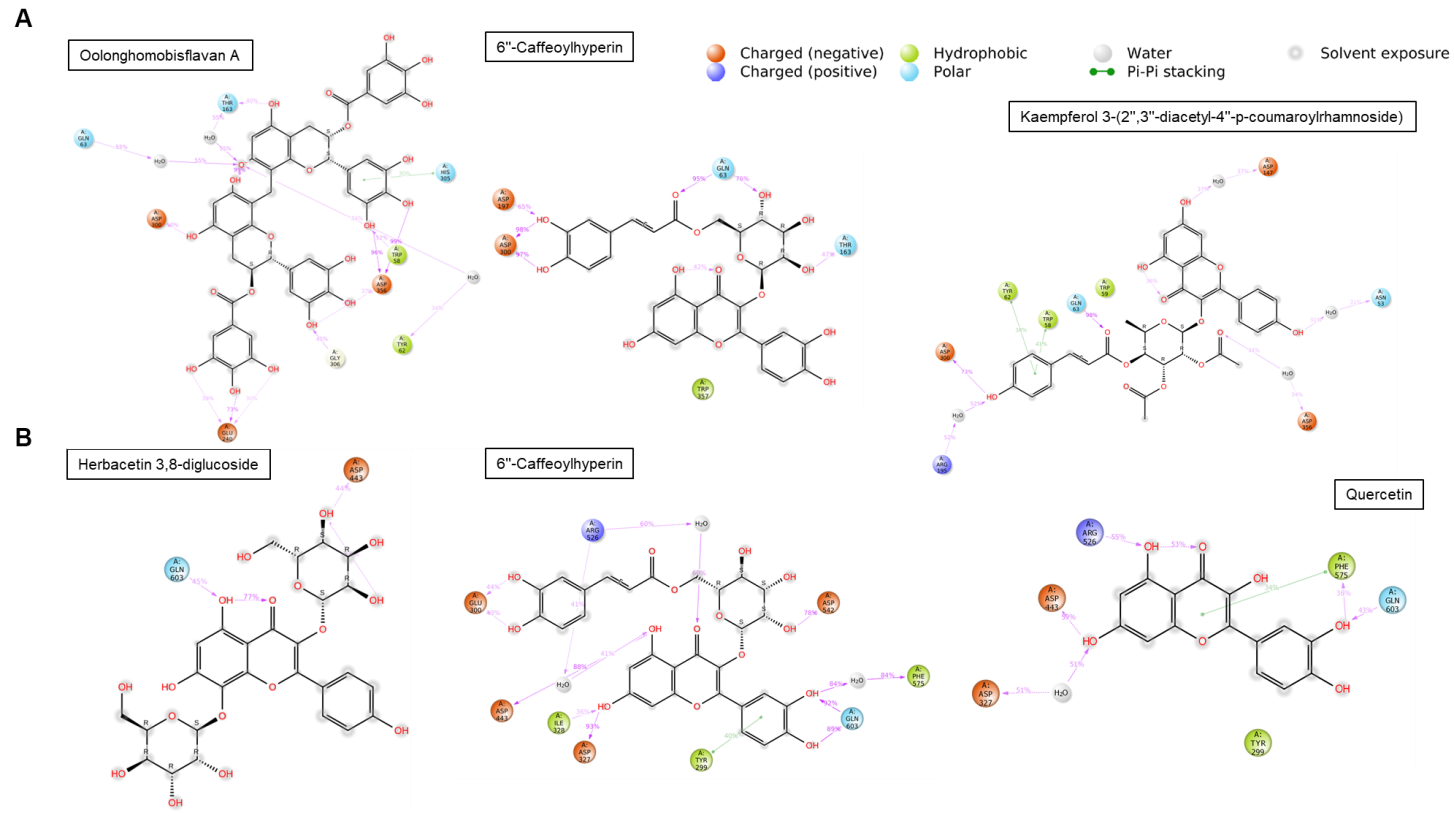
Interaction duration of different polyphenolic compounds with binding site residues of **(A)** human α-amylase (PDB: 1HNY) and **(B)** human α-glucosidase (PDB: 3L4Y).

**Figure 7:**
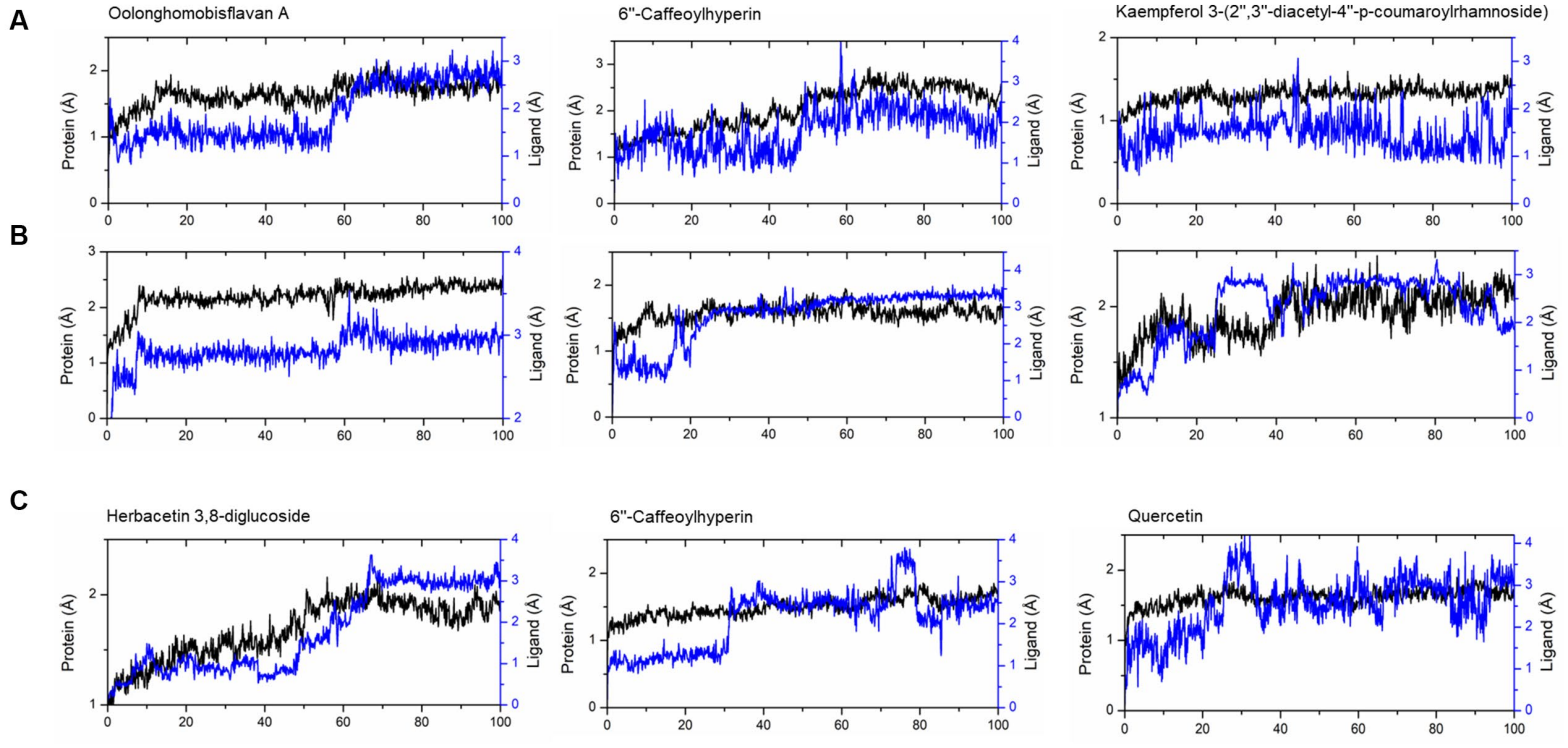
RMSD of trajectories calculated after molecular dynamic simulations of given ligand complexes with **(A)** human α-amylase (PDB: 1HNY), **(B)** porcine pancreatic α-amylase (PDB: 1OSE), **(C)** human α-glucosidase (PDB: 3L4Y).

**Figure 8:**
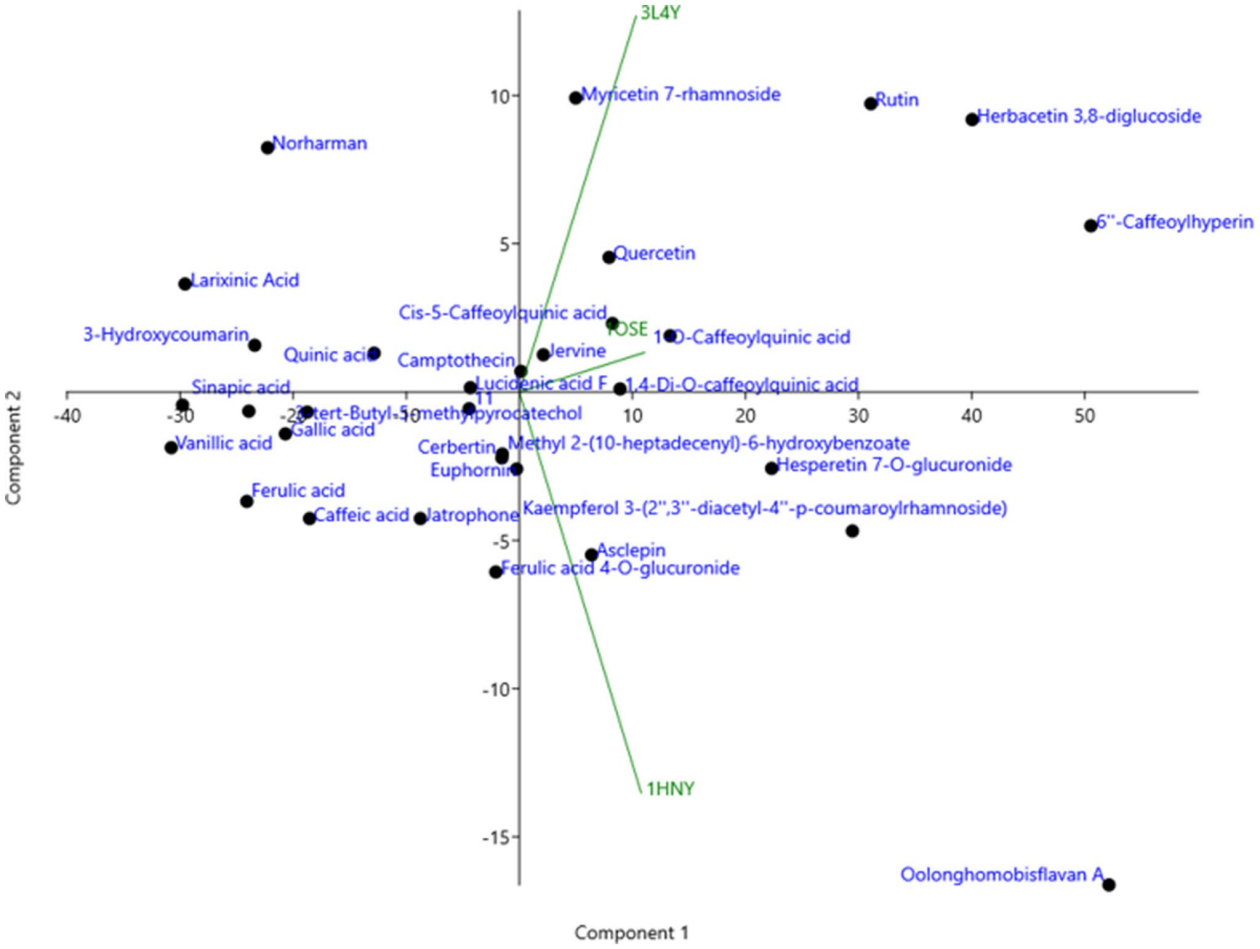
Principal component analysis (PCA) of binding energies of different polyphenol compounds identified in IA extract in complexed with different α-amylases and α-glucosidase represented by their PDB ids.

The compounds identified in LC-ESI-QTOF-MS and GC/MS analysis were evaluated for their interaction with different α-amylases (AML) and α-glucosidase (AGL). A set of 35 different compounds were selected resulting in a ligand library of 355 molecules after generation of tautomeric forms. The docking scores of all these molecules were listed in Supplementary Table S5 and the protein-ligand interaction of some compounds were shown in Figure 6. The stability of different interactions was represented by RMSD analysis of the MD simulation trajectory for 100 ns (Figure 7). Polyphenols showed a predominant interaction with AML and AGL enzymes, while a limited number of non-polyphenolic compounds such as jervine, asclepin and norharman also displayed significant levels of interaction with these enzymes. Oolonghomobisflavan A, 6″-caffeoylhyperin and herbacetin 3,8-diglucoside represented one of the strongest interactions for AMLs (Figure 6). Flavone glycosides such as rutin, herbacetin 3,8-diglucoside, myricetin 7-rhamnoside etc. represented strong docking score and binding energies for both AML and AGL. Moreover, caffeoylquinic acid derivatives and compounds like jervine and asclepin represented stronger docking score against AML and compounds like norharman for AGL. Although protein structures of human AML and PPA exhibited similar docking scores, the stability of similar compounds docked in the active sites varied nominally (Figure 7). This suggests that some compounds in IA extracts may be more effective at inhibiting human α-amylases as compared to PPA. For instance, kaempferol 3-(2″,3″-diacetyl-4″-p-coumaroylrhamnoside) may interact strongly and stably with human α-amylase as compared to PPA. The docking scores and binding energies of all the molecules were subjected to principal component analysis (Figure 8). PC1 comprised of total 90% variance and represented the compounds with or without significant interaction with α-amylases and α-glucosidase. PC2 comprised of 8% variance and distinguished the compounds that interacted with α-amylases and α-glucosidase. It was observed that flavone glucosides and rhamnosides interact strongly with AGL as compared to AML. However, 6″-caffeoylhyperin and caffeoylquinic acid derivatives were equally significant for interaction with both AML and AGL.

## 4 Discussion

IA is a wild aquatic plant, and its biochemical composition varies from region to region and across the cultivars. Moreover, there are different land races of IA available worldwide with several plant morphology. Recently, IA has gained specific attention with reports of its numerous bioactive properties such as anticancer, anti-hyperglycemic, anti-hyperlipidemic, prevention against hepatic injury etc. (3, 10, 29, 30). Within the few studied cultivars of IA, majority of them are from China and Malaysia, however, the cultivars originating from North East India are yet to be explored along with their biochemical composition as well as for medicinal values. Although IA has recently gained scientific attention, its complete nutritional and phytochemical composition is still not properly established and seems to vary significantly across different studies (3, 29). The present study suggests that IA is a rich source of minerals and fiber, which is in agreement with the previous works (31, 32). In comparison, average content of minerals in leafy vegetables is within 8 to 10% and also may be high upto 22% in case of non-conventional edible plants (5, 33); however, in our case the mineral content obtained was 14.99%. In addition, the relatively high content calcium and iron in IA is superior to many conventional leafy vegetables (34). Leafy vegetables are not considered as an abundant source of protein, in our case, protein content in IA was comparable to that of other vegetables. However, essential amino acid content was high as demonstrated by HPLC quantification as well as in GC–MS metabolomic analysis. By comparison, all major food grains contain total essential amino acids variably in the range of 33 to 43% (35), however, in case of IA it was higher upto 60%. Among the amino acid quantified IA exhibited promising amount of sulfur containing amino acid methionine and essential aromatic amino acid phenylalanine, which is of considerable nutritional benefit (36, 37). Furthermore, in the present study, IA exhibited the presence of important fatty acids, mainly α-linolenic acid and linolenic acid which are known to confer various health protecting properties. Contrary to the nutritional benefits of IA, certain wild *Ipomoea* species such as *Ipomoea carnea* and *Ipomoea batatas* have some anti nutritional properties due to presence of some toxic alkaloids (38, 39). However, such wild plants like *Ipomoea carnea* are not considered edible despite having some medicinal value. Likewise, for *Ipomoea batatas* as per traditional knowledge the tuber of the plant is edible while it is healthy and fully mature and at that stage these toxic alkaloids were found to be absent (38). However, traditional consumption of IA as leafy vegetable has been an age-old practice, and no such toxicities were known till date. Furthermore, the chemical composition revealed in the present work also substantiates absence of any such toxic compounds in the IA cultivar studied.

Along with its promising nutritional composition, IA also revealed the presence of substantial amounts of dietary antioxidants. IA exhibited significantly higher polyphenolic content (42.43 mg GAE/g dw) as compared to conventional leafy vegetables as well as many non-conventional food plants (4, 40, 41). Previous studies suggest that polyphenolic content in IA may be high upto 174.4 mg GAE/g dm which is equivalent to that of *Moringa oleifera* (42, 49). Flavonoids and many other polyphenolic compounds gained momentum with the reports that they are efficient antioxidants because of their strong ability to scavenge free radicals particularly reactive oxygen species (ROS) (46).

Several research have consistently shown that wild edible food plants, such as IA, possess a greater level of antioxidant activity in comparison to various leafy vegetables. In fruits and vegetables polyphenolic compounds as well as vitamins are majorly responsible for antioxidant activity. Polyphenols, particularly flavonoids and phenolic acids, are a considerable component of dietary antioxidants and are known for their remarkable antioxidant properties (3, 46). Furthermore, the phytochemical makeup of a plant can exhibit antioxidant activity synergistically in several mechanisms such as ROS scavenging, reducing metal ions as well as altering responses of innate enzymatic antioxidant mechanisms in human body (3). Along with high polyphenol content in IA, assessment of *in vitro* radical scavenging for both DPPH and ABTS revealed remarkable IC50 of inhibition indicating a significant potential to act on different ROS. In a recent work it was evidenced that glycoside merremoside from IA can exert anti breast cancer activity by regulation of ROS and altering responses of different enzymatic antioxidants in human body (3). Another key factor of antioxidant potential is reducing power of its constituents, in the present study both the FRAP assay and phosphomolybdate reduction capacity (TAC assay) revealed superior antioxidant capacity of IA as compared to many other food plants (5, 40, 41). Furthermore, prevention of ROS induced damage to biomolecules such as DNA is one of the major properties of antioxidant compounds. In the present study IA exhibited significant dose dependent oxidative DNA damage prevention (Figure 3) from hydroxyl radicals produced during UVC irradiation. This can be correlated directly to the free radical inhibition measurement by ORAC assay and strong radical scavenging activities exhibited by IA extracts. Similarly, a number of workers have demonstrated DNA damage prevention activity of plant extracts from other non-conventional food plants such as *Polygonum aviculare* (47), *Ludwigia octovalis*, *Bombax malabaricum*, (57) and *Curcuma species* (55).

In the present study, high resolution mass spectrometric identification of phytochemical composition revealed a significant proportion of polyphenolic compounds in IA (Tables 4, 5). Several identified compounds from IA in the present study were not reported in previous works. However, some studies have evidenced the presence of compounds such as 1-o-sinapoyl- β-D-glucose and derivatives of ferulic acids and caffeoylquinic acids (29, 49). Many of the listed compounds in the present are known to act as strong antioxidants in numerous *in vitro* and *in vivo* assays (48). Glycosides such as flavone glycosides comprise a dominant portion of IA phytochemical composition followed by phenolic acids. This corroborates with the previous reports and recently it was evidenced that extracted glycosides from IA can prevent some serious clinical conditions (3, 30). In the present study we have reported some polyphenol glycosides from IA such as herbacetin 3,8-diglucoside, myricetin 7-rhamnoside, hesperetin 7-O-glucuronide, ferulic acid 4-O-glucuronide and kaempferol 3-(2″,3″-diacetyl-4″-p-coumaroylrhamnoside). Hespertin glucuronide and its analogs are antioxidants known for their strong photoprotective activity, which may play a crucial role in prevention of UVC induced DNA damage (54). The significant genoprotective activity observed in the present study can be directly attributed to presence of such compounds. Derivatives of quinic acids and caffeoylquinic acids are important constituents of IA and other plants from genus *Ipomoea* (39, 56). Likewise, caffeic acid and chlorogenic acid are another compound which occurs in major quantities in IA and *Ipomoea asarifolia* which were also evidenced earlier (29, 49, 56). Apart from polyphenols several compounds such as euphornin, asclepin and jervine were found to be part of IA phytochemicals which are reported to be bioactive against hyperlipidemic, cancer and inflamatory activities (44, 50, 52). In addition, the present study quantifies 10 different polyphenolic compounds (Table 6) from IA, and to the best of our knowledge, this had not been carried out in any of the previous works.

Interest and studies on inhibition of digestive enzymes is growing because slowdown in hydrolysis of starch has immense curative effect on diabetes management through prevention of hyperglycemia (12, 45, 62). The present study examined the inhibitory effect of IA extract against PPA and YGU (Figures 4, 5) and revealed a stronger inhibition of YGU in comparison to acarbose while weakly inhibiting PPA. The ratio of inhibition between YGU and PPA is of utmost importance due to the potential consequences associated with a greater degree of α-amylase inhibition. Specifically, an elevated level of α-amylase inhibition may result in an increased amount of undigested starch, which in turn can lead to gastrointestinal issues such and an imbalanced microbiome (12, 43). Inhibition of PPA and YGU may occur in multiple ways, either the inhibitors compete directly with the enzyme or bind to the enzyme-substrate complex. Previous works demonstrate that different polyphenols exhibit different *Ki* values of PPA, YGU and their respective enzyme-substrate complexes (60, 63). In the present inhibition kinetic study, both PPA and YGU were inhibited by mixed inhibition mode which is due to the diverse phytochemical composition obtained in IA. In accordance to the dominant portion of polyphenol glycosides obtained in IA, numerous studies suggest plant extract rich in polyphenol glycosides can significantly inhibit α-amylase and downstream enzyme α-glucosidase in various kinetic modes (14, 45, 59). As an example, Chlorogenic acid, caffeic acid and compounds with caffeoyl substitution acts as mixed inhibitor against both PPA and YGU (58, 59); ferulic acid can inhibit PPA in mixed mode and inhibit YGU in non-competitive mode (63). In our case, the phytochemical makeup of IA revealed a significant quantity of such compounds, resulting in a mixed mode of inhibition kinetics, which includes both competitive and noncompetitive inhibitions.

The inhibition of AML and AGL by IA phytochemicals is primarily due to hydrogen bonds and hydrophobic interactions between the binding site residues and the ligand. Moreover, the electron domain that results from the C=C or C=O and aromatic ring exerts hydrophobic effect near the binding site residues resulting inhibitory effects (53). A similar phenomenon was observed with flavonoids and other compounds of IA interacting with AML and AGL amino acid residues (Figure 6). The increase in the number of hydroxyl groups in the polyphenolic compounds played an important role in AML and AGU inhibitions, which was also evidenced in earlier works (13, 53). Considering the amino acid interactions, OH groups in polyphenols interacted mainly with negatively charged polar residues *viz.* ASP and GLU through hydrogen bonding. Moreover, oxygens (C=O) interacted with polar residues mainly GLY, ASN, GLN, THR and HIS. Notably, ASP300, ASP356 and GLY60, hydrogen bond formation were strong and most frequently observed during MD simulation of the polyphenol complexes with human AML (Figure 6). Likewise in case of human AGL the same interactions were observed with ASP443 and GLN603. It is noteworthy that these residues are situated very close to the active site residues in case of both AML and AGU, strong interaction with which indicates greater chance of inhibiting the enzyme catalytic activity. In the present study, oolonghomobisflavan A was found to be strongly and stably interacting with of both human AML and PPA; studies suggest that occurrence of flavins and flavin like compounds results in significant inhibition of AML (13). Moreover, 6”-Caffeoylhyperin and caffeoylquinic acid derivatives comprised a significant portion of IA phytochemical composition and participated considerably in both AML and AGL interactions. Apart from phenolic compounds, a few compounds which were part of IA phytochemicals exhibited significant interaction with AML and AGL structures, this was shown in PCA analysis (Figure 8). Moreover, contrary to that there were compounds including certain phenolic acids which did not interacted considerably with the active site of AML and AGL, however, previous reports suggest such compounds can interact with substrates like starch or directly with enzyme-substrate complex and slow down the response of starch digestion (13, 61). Apart from polyphenols, other compounds including leaf polysaccharides and other carbohydrate derivatives were also reported to be inhibitors of AML and AGL (51). In the present study, metabolite profile of IA identified by GC/MS analysis revealed presence of such sugar derivatives and sugar alcohols which may also be active against PPA and YGU inhibitions.

## 5 Conclusion

The present study shows that IA is nutritionally rich with high amounts of minerals, dietary fiber and essential amino acids. It is also rich in dietary antioxidants *viz.* phenolics and flavonoids exhibiting high antioxidant activity. Thus, this has promising implication for health protective and health promoting purpose and also evidenced in recent works. Phytochemical composition revealed a dominant portion of flavone glycosides in IA followed by phenolic acids and some other compounds having pharmaceutical importance. The rich phytochemical makeup of IA revealed in the present study were previously unknown. Apart from identification of important secondary metabolites, the present study quantified 10 different polyphenol compounds with high redox potentiality, which implies a strong antioxidant potency of IA. The previous works on IA did not include quantitative determination of such polyphenolic compounds. The present study demonstrated that IA extract can considerably protect damage of DNA from free radicals, which can prevent the onset of many chronic and lifestyle diseases in humans. IA also exhibited a strong α-glucosidase and α-amylase inhibition potential with mixed inhibition kinetics. The inhibitory mechanism, kinetic parameters of inhibition along with the specific compounds present in IA responsible for such behavior were outlined in the present work. This phenomenon indicates that IA phytochemicals can prevent hyperglycemia, and this is very important for type II diabetes management. The study also suggests that the synergistic application of purified IA polyphenols with standard drugs like acarbose can bolster anti hyperglycemic effect on humans by similar inhibitory mechanisms outlined in the present study. In summary, IA as non-conventional wild leafy vegetable is nutritious as well as has remarkable nutraceutical value along with a diverse phytochemical composition which can impart numerous health protecting and health promoting properties.

## Data availability statement

The original contributions presented in the study are included in the article/Supplementary material, further inquiries can be directed to the corresponding authors.

## Author contributions

KS: Conceptualization, Data curation, Formal analysis, Investigation, Methodology, Visualization, Writing – original draft, Writing – review & editing. SD: Formal analysis, Methodology, Resources, Writing – review & editing. SH: Data curation, Formal analysis, Methodology, Writing – review & editing. GH: Methodology, Resources, Validation, Writing – review & editing. DT: Formal analysis, Investigation, Resources, Software, Writing – review & editing. AH: Supervision, Formal analysis, Investigation, Methodology, Validation, Writing – review & editing.
