# Supplementary material for: Chemical and biochemical characterization of Ipomoea aquatica: genoprotective potential and inhibitory mechanism of its phytochemicals against α-amylase and α-glucosidase
Source: Front Nutr. 2023 Dec 21;10:1304903. doi: 10.3389/fnut.2023.1304903 (PMC10772144; doi:10.3389/fnut.2023.1304903)
Supplement: Supplementary file 1 [file Data_Sheet_1.ZIP › supplementary/Supplementary Tables.docx]

**Supplementary table 1:** Analytical methods used for estimation of nutritional parameters.

| Analyte | Method | Reference |
| --- | --- | --- |
| Moisture | Oven drying method | Method 930.15 of AOAC |
| Crude protein | Microkjeldahl method | Method 2001.11 of AOAC |
| Lipid | Soxhlet extraction | Method 920.39 of AOAC |
| Crude fiber | Acid and Alkali hydrolysis | Method 962.09 of AOAC |
| Ash | Dry ashing | Method 942.05 of AOAC |
| Sodium | Dry ashing and Flame photometry | Modified method 968.08 of AOAC |
| Potassium | Dry ashing and Flame photometry | Modified method 968.08 of AOAC |
| Calcium | Wet ashing and AAS | Method 935.13 and 968.08 of AOAC |
| Magnesium | Wet ashing and AAS | Method 935.13 and 968.08 of AOAC |
| Manganese | Wet ashing and AAS | Method 935.13 and AAS |
| Iron | Dry ashing and AAS | Method 999.10 of AOAC |
| Zinc | Wet ashing and AAS | Method 999.10 of AOAC |
| Phosphorus | Gravimetric estimation | Method 964.06 of AOAC |

*AAS = atomic absorption spectrometry*

**Supplementary** **table 2:** List of proteins used for in silico interaction study.

|  | Uniprot ID | Protein name | PDB | Species |
| --- | --- | --- | --- | --- |
| 1 | P04746 | Pancreatic alpha-amylase | 1HNY | *Homo sapiens* |
| 2 | P00690 | Pancreatic alpha-amylase | 1OSE | *Sus scrofa* |
| 3 | O43451 | Maltase-glucoamylase | 3L4Y | *Homo sapiens* |
| 4 | P38138* | Glucosidase 2 subunit alpha | - | *Saccharomyces cerevisiae* |

* Homology models could not be built with high accuracy, hence hot considered in the present study.

**Supplementary table 3:** LC-ESI-qTOF-MS/MS determination of phytochemical composition of IA crude extract. Data acquired in +ESI mode.

| Sl no. | Name | Formula | Score | Mass | Base Peak | Polarity | RT | Algorithm |
| --- | --- | --- | --- | --- | --- | --- | --- | --- |
| 1 | Isoamyl nitrite | C_5_H_11_NO_2_ | 86.75 | 117.0786 | 118.0858 | Positive | 0.94 | Auto MS/MS |
| 2 | Retronecine | C_8_H_13_NO_2_ | 83.45 | 155.0938 | 118.0858 | Positive | 1.17 | Auto MS/MS |
| 3 | (R)-(+)-2-Pyrrolidone-5-carboxylic acid | C_5_H_7_NO_3_ | 93.78 | 129.0421 | 118.0857 | Positive | 1.24 | Auto MS/MS |
| 4 | L-isoleucyl-L-proline | C_11_H_20_N_2_O_3_ | 93.67 | 228.1462 | 229.1524 | Positive | 1.53 | Auto MS/MS |
| 5 | Larixinic Acid | C_6_H_6_O_3_ | 95.68 | 126.0309 | 127.0387 | Positive | 3.08 | Auto MS/MS |
| 6 | Indoleacrylic acid | C_11_H_9_NO_2_ | 96.09 | 187.0625 | 188.0694 | Positive | 3.17 | Auto MS/MS |
| 7 | Feruloyl-2-hydroxyputrescine | C_14_H_20_N_2_O_4_ | 79.88 | 280.1433 | 195.0894 | Positive | 4.09 | Auto MS/MS |
| 8 | L-1,2,3,4-Tetrahydro-beta-carboline-3-carboxylic acid | C_12_H_12_N_2_O_2_ | 79.78 | 216.0888 | 144.0798 | Positive | 4.30 | Auto MS/MS |
| 9 | Norharman | C_11_H_8_N_2_ | 79.25 | 168.0678 | 169.0751 | Positive | 4.84 | Auto MS/MS |
| 10 | 3-Hydroxycoumarin | C_9_H_6_O_3_ | 93.89 | 162.0306 | 163.0368 | Positive | 4.89 | Auto MS/MS |
| 11 | Solanocapsine | C_27_H_46_N_2_O_2_ | 76.2 | 430.3516 | 100.1109 | Positive | 5.54 | Auto MS/MS |
| 12 | Quercetin | C_15_H_10_O_7_ | 92.73 | 302.0412 | 303.0481 | Positive | 5.82 | Auto MS/MS |
| 13 | Hexyl 2-furoate | C_11_H_16_O_3_ | 95.67 | 196.109 | 197.1147 | Positive | 6.04 | Auto MS/MS |
| 14 | 2-Phenylethyl propanoate | C_11_H_14_O_2_ | 93.06 | 178.0983 | 163.036 | Positive | 6.12 | Auto MS/MS |
| 15 | Hesperetin 7-O-glucuronide | C_23_H_24_O_11_ | 98.96 | 476.1315 | 163.038 | Positive | 6.13 | Auto MS/MS |
| 16 | (S)-Edulinine | C_16_H_21_NO_4_ | 95.01 | 291.1475 | 177.0537 | Positive | 7.15 | Auto MS/MS |
| 17 | 3',4'-Dihydrodiol | C_15_H_14_N_2_O_4_ | 92.19 | 286.0964 | 263.0794 | Positive | 7.66 | Auto MS/MS |
| 18 | Dihydrocapsaicin | C_18_H_29_NO_3_ | 89.4 | 307.213 | 308.2203 | Positive | 7.91 | Auto MS/MS |
| 19 | 16-Oxo-palmitate | C_16_H_30_O_3_ | 86.66 | 270.2204 | 275.1971 | Positive | 8.75 | Auto MS/MS |
| 20 | Geranyl 2-ethylbutyrate | C_16_H_28_O_2_ | 86.5 | 252.21 | 275.198 | Positive | 8.99 | Auto MS/MS |
| 21 | 3-tert-Butyl-5-methylcatechol | C_11_H_16_O_2_ | 93.56 | 180.1139 | 181.1212 | Positive | 9.39 | Auto MS/MS |
| 22 | (9Z,11E,13E,15Z)-4-Oxo-9,11,13,15-octadecatetraenoic acid | C_18_H_26_O_3_ | 88.81 | 290.1872 | 291.1927 | Positive | 10.30 | Auto MS/MS |
| 23 | 2-(3-Phenylpropyl) tetrahydrofuran | C_13_H_18_O | 94.9 | 190.1348 | 191.1419 | Positive | 11.70 | Auto MS/MS |
| 24 | Cerbertin | C_32_H_44_O_11_ | 97.63 | 604.2891 | 567.2552 | Positive | 12.06 | Auto MS/MS |
| 25 | Citronellyl hexanoate | C_16_H_30_O_2_ | 96.24 | 254.2255 | 277.2155 | Positive | 13.18 | Auto MS/MS |
| 26 | Myxalamid B | C_25_H_39_NO_3_ | 99.32 | 401.293 | 120.0793 | Positive | 13.42 | Auto MS/MS |
| 27 | Palmitic Acid | C_16_H_32_O_2_ | 95.97 | 256.241 | 279.2297 | Positive | 13.81 | Auto MS/MS |
| 28 | Asclepin | C_31_H_42_O_10_ | 96.91 | 574.2785 | 503.2405 | Positive | 16.08 | Auto MS/MS |
| 29 | Camptothecin derivative | C_20_H_16_N_2_O_4_ | 96.48 | 360.4491 | 362.8563 | Positive | 16.83 | Auto MS/MS |
| 30 | Ganosporelactone A | C_30_H_40_O_7_ | 95.74 | 512.2784 | 535.2679 | Positive | 18.15 | Auto MS/MS |
| 31 | Ganoderic acid F | C_32_H_42_O_9_ | 98.01 | 570.2837 | 533.2517 | Positive | 18.23 | Auto MS/MS |
| 32 | 20-Hydroxy-3,7,11,15,23-pentaoxolanost-8-en-26-oic acid | C_30_H_40_O_8_ | 91.31 | 528.2736 | 551.2619 | Positive | 18.92 | Auto MS/MS |
| 33 | Euphornin | C_33_H_44_O_9_ | 97.48 | 584.2992 | 547.2683 | Positive | 19.17 | Auto MS/MS |
| 34 | Methyl 2-(10-heptadecenyl)-6-hydroxybenzoate | C_25_H_40_O_3_ | 99.74 | 388.2977 | 389.30501 | Positive | 20.33 | Auto MS/MS |

**Supplementary table 4:** LC-ESI-qTOF-MS/MS determination of phytochemical composition of IA crude extract. Data acquired in -ESI mode.

| Sl no. | Name | Formula | Score | Mass | Base Peak | Polarity | RT | Algorithm |
| --- | --- | --- | --- | --- | --- | --- | --- | --- |
| 1 | Quinic acid | C_7_H_12_O_6_ | 93.42 | 192.0622 | 191.0552 | Negative | 1.08 | Auto MS/MS |
| 2 | L-Malic acid | C_4_H_6_O_5_ | 90 | 134.0206 | 133.0123 | Negative | 1.15 | Auto MS/MS |
| 3 | 2,5-Didehydro-D-gluconate | C_6_H_8_O_7_ | 94.78 | 192.026 | 111.0074 | Negative | 1.31 | Auto MS/MS |
| 4 | Gallic acid | C_7_H_6_O_5_ | 78.21 | 170.1213 | 169.0137 | Negative | 1.70 | Auto MS/MS |
| 5 | Methylisocitric acid | C_7_H_10_O_7_ | 95.17 | 206.0418 | 111.0071 | Negative | 2.80 | Auto MS/MS |
| 6 | 1-O-Caffeoylquinic acid | C_16_H_18_O_9_ | 94.8 | 354.0939 | 191.0555 | Negative | 3.08 | Auto MS/MS |
| 7 | Cis-5-Caffeoylquinic acid | C_16_H_18_O_9_ | 93.52 | 354.0936 | 191.055 | Negative | 4.00 | Auto MS/MS |
| 8 | Caffeic acid | C_9_H_8_O_4_ | 92.98 | 180.0411 | 135.0433 | Negative | 4.43 | Auto MS/MS |
| 9 | Phenylacetic acid | C_8_H_8_O_2_ | 81.09 | 136.0514 | 179.0344 | Negative | 4.54 | Auto MS/MS |
| 10 | Herbacetin 3,8-diglucoside | C_27_H_30_O_17_ | 95.81 | 626.1467 | 300.0255 | Negative | 5.08 | Auto MS/MS |
| 11 | (1xi,3xi)-1,2,3,4-Tetrahydro-1-methyl-beta-carboline-3-carboxylic acid | C_13_H_14_N_2_O_2_ | 91.36 | 230.1047 | 127.0502 | Negative | 5.40 | Auto MS/MS |
| 12 | Vanillic acid derivative | C_8_H_8_O_4_ | 90.36 | 168.1531 | 167.0344 | Negative | 5.51 | Auto MS/MS |
| 13 | 4-Hydroxyphenylacetylglutamine | C_13_H_15_NO_6_ | 95.9 | 281.0889 | 164.0711 | Negative | 5.58 | Auto MS/MS |
| 14 | Myricetin 7-rhamnoside | C_21_H_20_O_12_ | 97.49 | 464.0946 | 300.0262 | Negative | 5.77 | Auto MS/MS |
| 15 | Oolonghomobisflavan A | C_45_H_36_O_22_ | 75.53 | 928.1902 | 928.1698 | Negative | 5.84 | Auto MS/MS |
| 16 | Chlorogenic acid | C_16_H_18_O_9_ | 76.41 | 354.312 | 335.0765 | Negative | 6.38 | Auto MS/MS |
| 17 | 1,4-Di-O-caffeoylquinic acid | C_25_H_24_O_12_ | 98.18 | 516.1262 | 179.0351 | Negative | 6.69 | Auto MS/MS |
| 18 | 6-(Pentylthio)purine | C_10_H_14_N_4_S | 84.91 | 222.0923 | 267.0904 | Negative | 7.02 | Auto MS/MS |
| 19 | 6''-Caffeoylhyperin | C_30_H_26_O_15_ | 99.77 | 626.1273 | 300.026 | Negative | 7.46 | Auto MS/MS |
| 20 | Kaempferol 3-(2'',3''-diacetyl-4''-p-coumaroylrhamnoside) | C_34_H_30_O_14_ | 99.78 | 662.1638 | 191.0553 | Negative | 7.47 | Auto MS/MS |
| 21 | Rutin | C_27_H_30_O_16_ | 87.63 | 610.5231 | 457.1354 | Negative | 7.68 | Auto MS/MS |
| 22 | Quercetin-3β-D-glucoside | C_21_H_20_O_12_ | 86.14 | 464.4132 | 301.0378 | Negative | 8.18 | Auto MS/MS |
| 23 | Corchorifatty acid F | C_18_H_32_O_5_ | 99.08 | 328.2249 | 327.2167 | Negative | 8.73 | Auto MS/MS |
| 24 | Ferulic acid | C_10_H_10_O_4_ | 75.23 | 194.1812 | 193.0501 | Negative | 8.91 | Auto MS/MS |
| 25 | 9,10-Dihydroxy-12,13-epoxyoctadecanoate | C_18_H_34_O_5_ | 99.11 | 330.2402 | 329.2333 | Negative | 9.21 | Auto MS/MS |
| 26 | Sinapic acid | C_11_H_12_O_5_ | 83.24 | 224.2101 | 223.0105 | Negative | 11.23 | Auto MS/MS |
| 27 | 9Z-Octadecenedioic acid | C_18_ H_32_O_4_ | 97.58 | 312.2292 | 311.2213 | Negative | 11.38 | Auto MS/MS |
| 28 | Sphinganine-phosphate | C_18_H_40_NO_5_P | 89.92 | 381.2657 | 164.0706 | Negative | 13.34 | Auto MS/MS |
| 29 | Lucidenic acid F | C_27_H_36_O_6_ | 99.46 | 456.251 | 134.0378 | Negative | 14.65 | Auto MS/MS |
| 30 | Ferulic acid 4-O-glucuronide | C_16_H_18_O_10_ | 78.29 | 370.3152 | 369.261 | Negative | 15.29 | Auto MS/MS |
| 31 | 17beta-[Bis(2-hydroxyethyl)amino]androst-5-en-3beta-ol | C_23_H_39_NO_3_ | 99.03 | 377.2926 | 116.0715 | Negative | 16.08 | Auto MS/MS |
| 32 | Jervine | C_27_H_39_NO_3_ | 80.99 | 425.2918 | 164.0714 | Negative | 16.59 | Auto MS/MS |
| 33 | Geranylfarnesyl diphosphate | C_25_H_44_O_7_P_2_ | 86.71 | 518.2543 | 577.2673 | Negative | 16.67 | Auto MS/MS |
| 34 | Methyl 2-(10-heptadecenyl)-6-hydroxybenzoate | C_25_H_40_O_3_ | 99.74 | 388.2978 | 134.8943 | Negative | 20.33 | Auto MS/MS |
| 35 | Jatrophone | C_20_H_24_O_3_ | 87.17 | 312.1734 | 311.1666 | Negative | 26.72 | Auto MS/MS |

**Supplementary table 5:** Molecular docking scores and binding energies of different compounds identified from IA extract with different AML and AGU proteins represented by their PDB IDs.

| Sl no. | Compound name | PubChem CID | Docking scores of protein complexes | | | Binding energies of protein complexes (kcal/mol) | | |
| --- | --- | --- | --- | --- | --- | --- | --- | --- |
|  |  |  | **1HNY** | **1OSE** | **3L4Y** | **1HNY** | **1OSE** | **3L4Y** |
| 1 | Oolonghomobisflavan A | 14520989 | -9.36 | -7.63 | -4.99 | -82.67 | -69.99 | -55.45 |
| 2 | 6''-Caffeoylhyperin | 131752204 | -8.44 | -8.20 | -7.64 | -66.78 | -68.03 | -71.28 |
| 3 | Kaempferol 3-(2'',3''-diacetyl-4''-p-coumaroylrhamnoside) | 14630678 | -8.30 | -7.39 | -5.20 | -60.46 | -58.16 | -50.50 |
| 4 | Herbacetin 3,8-diglucoside | 14375133 | -7.68 | -8.39 | -6.86 | -53.85 | -71.26 | -62.40 |
| 5 | Hesperetin 7-O-glucuronide | 71777476 | -7.42 | -6.53 | -4.41 | -55.05 | -53.46 | -48.32 |
| 6 | Caffeic acid | 689043 | -6.69 | -5.71 | -4.78 | -32.39 | -29.42 | -24.22 |
| 7 | Gallic acid | 370 | -6.55 | -5.53 | -5.17 | -29.16 | -28.16 | -25.09 |
| 8 | 1-O-Caffeoylquinic acid | 10155076 | -6.51 | -7.21 | -5.59 | -43.94 | -54.22 | -42.95 |
| 9 | Rutin | 5280805 | -6.42 | -4.96 | -7.39 | -55.67 | -49.82 | -67.38 |
| 10 | Quinic acid | 6508 | -6.32 | -6.14 | -5.07 | -30.73 | -35.21 | -30.01 |
| 11 | (S)-Edulinine | 356087 | -6.31 | -6.64 | -3.74 | -36.95 | -40.12 | -33.38 |
| 12 | Camptothecin | 24360 | -6.30 | -5.57 | -5.01 | -39.26 | -41.67 | -37.53 |
| 13 | Quercetin | 5280343 | -6.28 | -7.79 | -6.36 | -40.23 | -48.26 | -43.50 |
| 14 | 3-tert-Butyl-5-methylpyrocatechol | 66095 | -6.09 | -5.32 | -5.26 | -29.97 | -28.88 | -26.95 |
| 15 | Ferulic acid 4-O-glucuronide | 6443140 | -6.08 | -6.17 | -2.91 | -42.54 | -40.66 | -31.21 |
| 16 | 3-Hydroxycoumarin | 13650 | -5.94 | -4.66 | -6.25 | -26.72 | -23.90 | -27.31 |
| 17 | Jervine | 10098 | -5.77 | -6.03 | -4.20 | -39.68 | -43.61 | -38.60 |
| 18 | Ferulic acid | 445858 | -5.76 | -4.41 | -4.49 | -29.73 | -24.03 | -22.79 |
| 19 | Lucidenic acid F | 23247893 | -5.57 | -4.49 | -4.21 | -37.67 | -37.68 | -35.44 |
| 20 | Jatrophone | 5281373 | -5.49 | -5.61 | -2.73 | -35.48 | -40.90 | -26.31 |
| 21 | Vanillic acid | 8468 | -5.42 | -5.90 | -4.57 | -23.91 | -21.55 | -19.51 |
| 22 | 4-Hydroxybenzoic acid | 135 | -5.36 | -5.40 | -4.90 | -22.20 | -24.95 | -19.43 |
| 23 | Norharman | 64961 | -5.31 | -6.20 | -6.56 | -21.25 | -27.88 | -30.82 |
| 24 | Cis-5-Caffeoylquinic acid | 1794425 | -5.26 | -6.44 | -3.98 | -42.52 | -47.20 | -42.78 |
| 25 | Myricetin 7-rhamnoside | 73419 | -5.21 | -7.30 | -4.99 | -34.04 | -48.13 | -44.79 |
| 26 | Asclepin | 441844 | -5.17 | -4.43 | - | -47.82 | -44.02 | - |
| 27 | Sinapic acid | 637775 | -5.06 | -4.85 | -3.57 | -27.10 | -25.45 | -24.31 |
| 28 | 1,4-Di-O-caffeoylquinic acid | 1794427 | -4.92 | -7.46 | -4.63 | -42.64 | -51.52 | -39.23 |
| 29 | Larixinic Acid | 8369 | -4.74 | -4.72 | -4.47 | -21.10 | -21.56 | -24.61 |
| 30 | Euphornin | 6440619 | -4.45 | -3.49 | -3.35 | -44.44 | -34.67 | -38.96 |
| 31 | Cerbertin | 441851 | -3.73 | -4.65 | - | -42.52 | -35.88 | - |
| 32 | Methyl 2-(10-heptadecenyl)-6-hydroxybenzoate | 131752389 | -3.54 | -4.11 | -2.01 | -40.47 | -40.22 | -34.88 |

**Supplementary table 6:** Parameters of HPLC method used for quantification of 10 polyphenolic compounds.

|  | Compound | **Wavelength** | **RT (min)** | **Slope** | **Intercept** | **R^2^** | **LOD (μg/mL)** | **LOQ (μg/mL)** |
| --- | --- | --- | --- | --- | --- | --- | --- | --- |
| 1 | Gallic acid | 255 | 2.83 | 4.2E-08 | 0.0027 | 0.998 | 9.43 | 15.26 |
|  |  | 280 |  | 2.8E-08 | 0.0012 | 0.996 | 3.06 | 4.80 |
| 2 | Chlorogenic acid | 280 | 3.61 | 2.9E-08 | 0.0003 | 0.999 | 1.61 | 2.79 |
|  |  | 255 |  | 4.3E-08 | 0.0003 | 0.998 | 2.94 | 5.39 |
| 3 | 4-hydroxy benzoic acid | 255 | 6.51 | 3.0E-08 | 0.0001 | 0.998 | 3.27 | 6.17 |
|  |  | 280 |  | 2.3E-08 | 0.0012 | 0.999 | 3.55 | 5.75 |
|  |  | 320 |  | 1.1E-08 | 0.0008 | 0.998 | 4.08 | 7.03 |
| 4 | Vanillic acid | 255 | 7.39 | 6.7E-09 | 0.0002 | 0.997 | 1.45 | 2.66 |
|  |  | 280 |  | 2.8E-08 | 0.0000 | 0.998 | 1.11 | 2.15 |
| 5 | Caffeic acid | 280 | 7.92 | 1.6E-09 | 0.0082 | 0.997 | 0.29 | 2.90 |
|  |  | 320 |  | 1.4E-08 | 0.0052 | 0.998 | 0.32 | 2.07 |
| 6 | Rutin | 255 | 9.31 | 2.3E-08 | 0.0004 | 0.998 | 1.87 | 3.26 |
|  |  | 280 |  | 5.9E-08 | 0.0004 | 0.998 | 1.54 | 2.56 |
|  |  | 320 |  | 4.3E-08 | 0.0003 | 0.999 | 1.35 | 2.34 |
|  |  | 375 |  | 3.8E-08 | 0.0003 | 0.999 | 1.60 | 2.81 |
| 7 | sinapic acid | 255 | 9.91 | 5.3E-08 | 0.0003 | 0.998 | 1.94 | 3.45 |
|  |  | 280 |  | 9.9E-09 | 0.0002 | 0.997 | 1.22 | 2.19 |
|  |  | 320 |  | 7.3E-09 | 0.0002 | 0.998 | 1.24 | 2.20 |
| 8 | Ferulic acid | 255 | 10.98 | 7.9E-09 | 0.0001 | 0.999 | 1.27 | 2.34 |
|  |  | 280 |  | 5.1E-09 | 0.0005 | 0.998 | 2.27 | 3.89 |
|  |  | 320 |  | 2.5E-09 | 0.0002 | 0.998 | 1.34 | 2.41 |
|  |  | 375 |  | 2.0E-07 | -0.0013 | 0.999 | 4.53 | 9.67 |
| 9 | Naringin | 280 | 10.99 | 1.5E-08 | -0.0008 | 0.999 | 4.76 | 9.69 |
| 10 | Quercetin | 255 | 14.81 | 2.6E-08 | 0.0005 | 0.998 | 2.06 | 3.48 |
|  |  | 280 |  | 7.4E-08 | 0.0011 | 0.999 | 2.56 | 3.96 |
|  |  | 320 |  | 6.5E-08 | 0.0011 | 0.999 | 3.02 | 4.81 |
|  |  | 375 |  | 2.6E-08 | 0.0013 | 0.999 | 3.46 | 5.47 |
