# Supplementary material for: Chemical and biochemical characterization of Ipomoea aquatica: genoprotective potential and inhibitory mechanism of its phytochemicals against α-amylase and α-glucosidase
Source: Front Nutr. 2023 Dec 21;10:1304903. doi: 10.3389/fnut.2023.1304903 (PMC10772144; doi:10.3389/fnut.2023.1304903)
Supplement: Supplementary file 1 [file Data_Sheet_1.ZIP › supplementary/Supplementary Figures.docx]

**
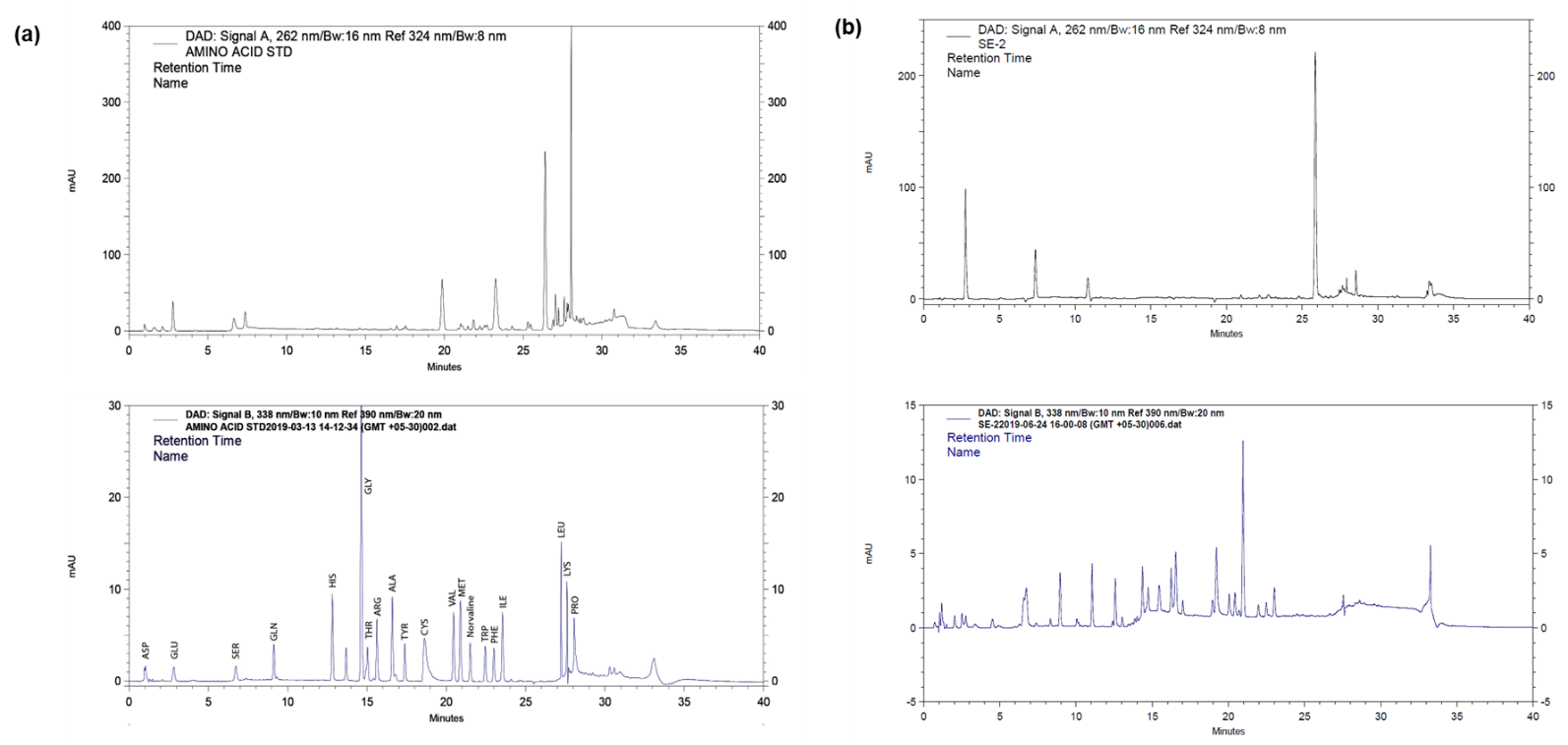
**

**Supplementary figure 1:** HPLC separation chromatogram of individual amino acids in (a) amino acid standard mixture and (b) IA. Top – secondary amino acids; bottom – primary amino acids.

**

**

**Supplementary figure 2:** Total ion chromatogram obtained in GC-MS analysis of derivatized IA crude extract.
