# Supplementary material for: Chemical and biochemical characterization of Ipomoea aquatica: genoprotective potential and inhibitory mechanism of its phytochemicals against α-amylase and α-glucosidase
Source: Front Nutr. 2023 Dec 21;10:1304903. doi: 10.3389/fnut.2023.1304903 (PMC10772144; doi:10.3389/fnut.2023.1304903)
Supplement: Supplementary file 1 [file Data_Sheet_1.ZIP › supplementary/Methods.docx]

**Materials and methods**

## Amino acid quantification using HPLC-DAD

Extraction of amino acids was carried out according to method 994.12 of AOAC (AOAC, 2016) with some modifications (Lamp, Kaltschmitt, & Ludtke, 2018). 100 mg of finely ground dry matter was oxidized at 0^o^C for 16 h min using 5 mL of performic acid solution (0.5 mL 30 % hydrogen peroxide, 3.88 mL formic acid and 0.6 mL water containing 0.02 g crystalline phenol). This was followed by the addition of 0.84 g sodium metabisulfite and 50 mL 6M HCl-phenol and hydrolyzed for 24 h at 110^o^C with periodic stirring. The hydrolysate was allowed to cool to room temperature and pH was adjusted to 2.20 using NaOH solution. An aliquot of 5 mL was filtered and used for HPLC analysis.

Quantification of amino acids was carried out in an Agilent 1260 HPLC system (Agilent, USA) with Agilent Zorbax Hypersil AA-ODS column, 2.1 x 200 mm (ID x L), 5 μm particle size and detected using a diode array detector. Mobile phases comprised of 40 mM Phosphate buffer, pH 7.8 (A) and Methanol/Acetonitrile/Water, 45:45:10 (B). A gradient program of 2%B for 0 min and 2.5 min, 57%B for 24.1 min, 100%B for 24.8 min and 29.7 min, 2%B for 40 min was used for separation with flow rate 0.5 mL/min and column temperature at 40^o^C. Pre-column derivatization was conducted using FMOC and OPA reagent followed by 20 μL injection using auto sampler.
