# Supplementary figures and images for: Chemical and biochemical characterization of Ipomoea aquatica: genoprotective potential and inhibitory mechanism of its phytochemicals against α-amylase and α-glucosidase (part 2 of 2)
Source: Front Nutr. 2023 Dec 21;10:1304903. doi: 10.3389/fnut.2023.1304903 (PMC10772144; doi:10.3389/fnut.2023.1304903)

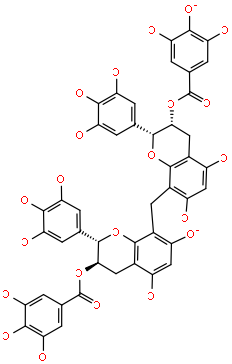

Supplement: Supplementary file 1 [file Data_Sheet_1.ZIP › supplementary/ligands-with-tautomers/structures/mol-112.png]

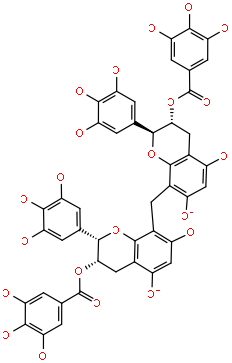

Supplement: Supplementary file 1 [file Data_Sheet_1.ZIP › supplementary/ligands-with-tautomers/structures/mol-106.png]

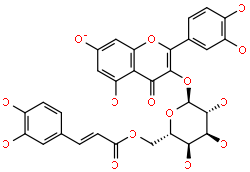

Supplement: Supplementary file 1 [file Data_Sheet_1.ZIP › supplementary/ligands-with-tautomers/structures/mol-338.png]

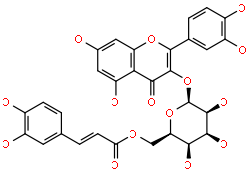

Supplement: Supplementary file 1 [file Data_Sheet_1.ZIP › supplementary/ligands-with-tautomers/structures/mol-310.png]

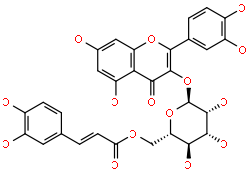

Supplement: Supplementary file 1 [file Data_Sheet_1.ZIP › supplementary/ligands-with-tautomers/structures/mol-304.png]

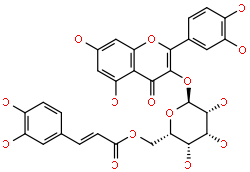

Supplement: Supplementary file 1 [file Data_Sheet_1.ZIP › supplementary/ligands-with-tautomers/structures/mol-305.png]

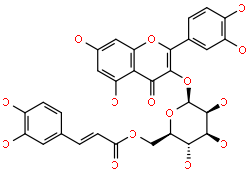

Supplement: Supplementary file 1 [file Data_Sheet_1.ZIP › supplementary/ligands-with-tautomers/structures/mol-311.png]

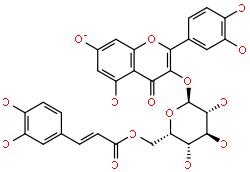

Supplement: Supplementary file 1 [file Data_Sheet_1.ZIP › supplementary/ligands-with-tautomers/structures/mol-339.png]

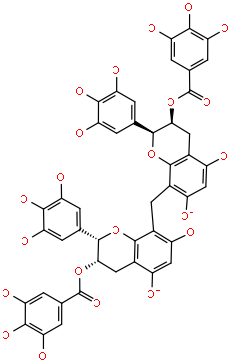

Supplement: Supplementary file 1 [file Data_Sheet_1.ZIP › supplementary/ligands-with-tautomers/structures/mol-107.png]

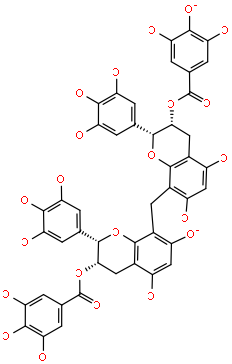

Supplement: Supplementary file 1 [file Data_Sheet_1.ZIP › supplementary/ligands-with-tautomers/structures/mol-113.png]

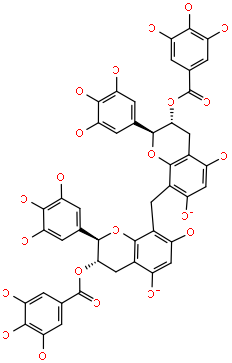

Supplement: Supplementary file 1 [file Data_Sheet_1.ZIP › supplementary/ligands-with-tautomers/structures/mol-098.png]

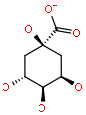

Supplement: Supplementary file 1 [file Data_Sheet_1.ZIP › supplementary/ligands-with-tautomers/structures/mol-073.png]

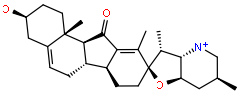

Supplement: Supplementary file 1 [file Data_Sheet_1.ZIP › supplementary/ligands-with-tautomers/structures/mol-067.png]

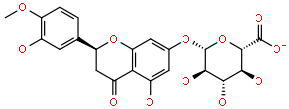

Supplement: Supplementary file 1 [file Data_Sheet_1.ZIP › supplementary/ligands-with-tautomers/structures/mol-271.png]

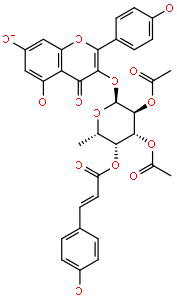

Supplement: Supplementary file 1 [file Data_Sheet_1.ZIP › supplementary/ligands-with-tautomers/structures/mol-265.png]

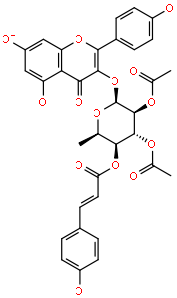

Supplement: Supplementary file 1 [file Data_Sheet_1.ZIP › supplementary/ligands-with-tautomers/structures/mol-259.png]

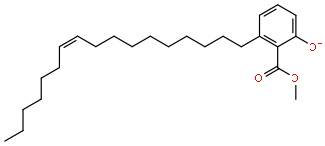

Supplement: Supplementary file 1 [file Data_Sheet_1.ZIP › supplementary/ligands-with-tautomers/structures/mol-202.png]

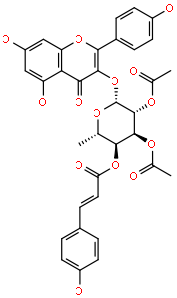

Supplement: Supplementary file 1 [file Data_Sheet_1.ZIP › supplementary/ligands-with-tautomers/structures/mol-216.png]

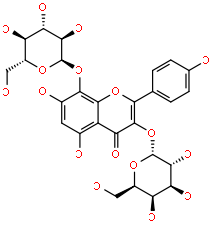

Supplement: Supplementary file 1 [file Data_Sheet_1.ZIP › supplementary/ligands-with-tautomers/structures/mol-014.png]

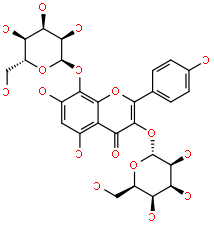

Supplement: Supplementary file 1 [file Data_Sheet_1.ZIP › supplementary/ligands-with-tautomers/structures/mol-028.png]

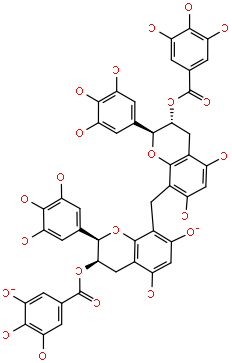

Supplement: Supplementary file 1 [file Data_Sheet_1.ZIP › supplementary/ligands-with-tautomers/structures/mol-174.png]

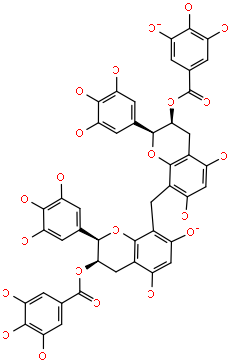

Supplement: Supplementary file 1 [file Data_Sheet_1.ZIP › supplementary/ligands-with-tautomers/structures/mol-160.png]

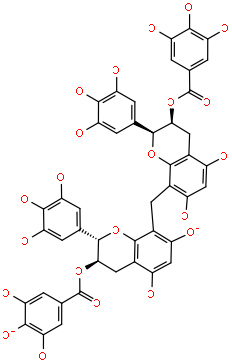

Supplement: Supplementary file 1 [file Data_Sheet_1.ZIP › supplementary/ligands-with-tautomers/structures/mol-148.png]

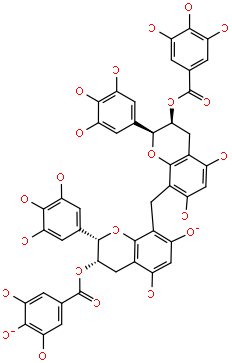

Supplement: Supplementary file 1 [file Data_Sheet_1.ZIP › supplementary/ligands-with-tautomers/structures/mol-149.png]

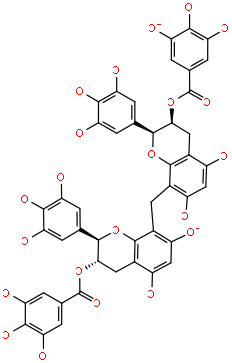

Supplement: Supplementary file 1 [file Data_Sheet_1.ZIP › supplementary/ligands-with-tautomers/structures/mol-161.png]

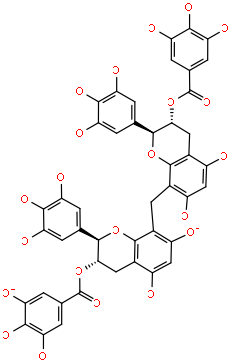

Supplement: Supplementary file 1 [file Data_Sheet_1.ZIP › supplementary/ligands-with-tautomers/structures/mol-175.png]

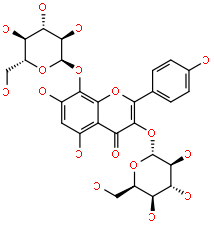

Supplement: Supplementary file 1 [file Data_Sheet_1.ZIP › supplementary/ligands-with-tautomers/structures/mol-029.png]

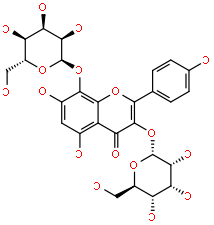

Supplement: Supplementary file 1 [file Data_Sheet_1.ZIP › supplementary/ligands-with-tautomers/structures/mol-015.png]

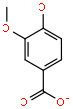

Supplement: Supplementary file 1 [file Data_Sheet_1.ZIP › supplementary/ligands-with-tautomers/structures/mol-001.png]

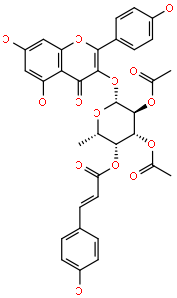

Supplement: Supplementary file 1 [file Data_Sheet_1.ZIP › supplementary/ligands-with-tautomers/structures/mol-217.png]

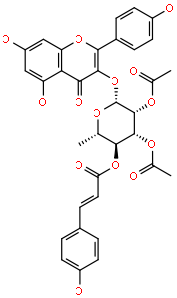

Supplement: Supplementary file 1 [file Data_Sheet_1.ZIP › supplementary/ligands-with-tautomers/structures/mol-215.png]

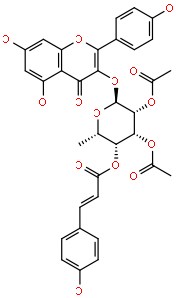

Supplement: Supplementary file 1 [file Data_Sheet_1.ZIP › supplementary/ligands-with-tautomers/structures/mol-229.png]

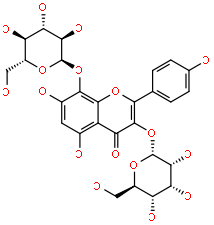

Supplement: Supplementary file 1 [file Data_Sheet_1.ZIP › supplementary/ligands-with-tautomers/structures/mol-017.png]

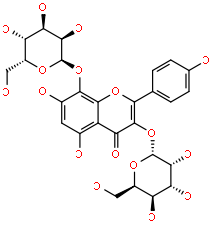

Supplement: Supplementary file 1 [file Data_Sheet_1.ZIP › supplementary/ligands-with-tautomers/structures/mol-003.png]

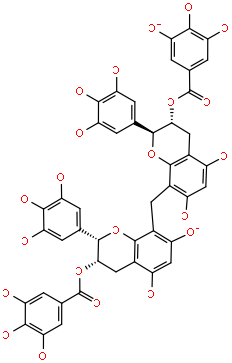

Supplement: Supplementary file 1 [file Data_Sheet_1.ZIP › supplementary/ligands-with-tautomers/structures/mol-163.png]

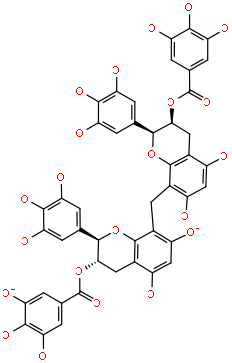

Supplement: Supplementary file 1 [file Data_Sheet_1.ZIP › supplementary/ligands-with-tautomers/structures/mol-177.png]

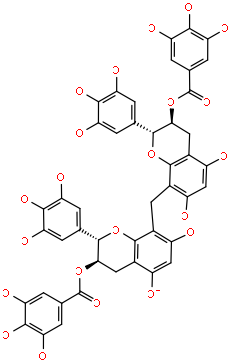

Supplement: Supplementary file 1 [file Data_Sheet_1.ZIP › supplementary/ligands-with-tautomers/structures/mol-188.png]

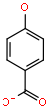

Supplement: Supplementary file 1 [file Data_Sheet_1.ZIP › supplementary/ligands-with-tautomers/structures/mol-348.png]

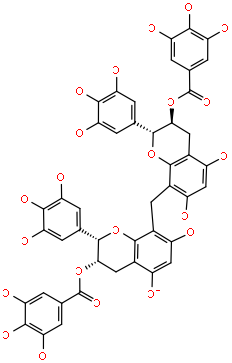

Supplement: Supplementary file 1 [file Data_Sheet_1.ZIP › supplementary/ligands-with-tautomers/structures/mol-189.png]

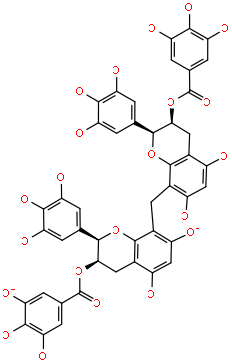

Supplement: Supplementary file 1 [file Data_Sheet_1.ZIP › supplementary/ligands-with-tautomers/structures/mol-176.png]

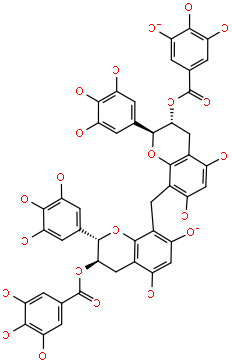

Supplement: Supplementary file 1 [file Data_Sheet_1.ZIP › supplementary/ligands-with-tautomers/structures/mol-162.png]

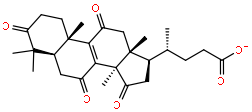

Supplement: Supplementary file 1 [file Data_Sheet_1.ZIP › supplementary/ligands-with-tautomers/structures/mol-002.png]

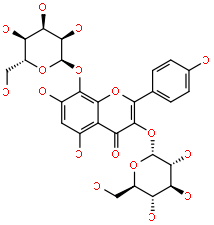

Supplement: Supplementary file 1 [file Data_Sheet_1.ZIP › supplementary/ligands-with-tautomers/structures/mol-016.png]

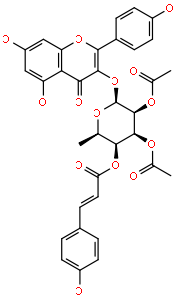

Supplement: Supplementary file 1 [file Data_Sheet_1.ZIP › supplementary/ligands-with-tautomers/structures/mol-228.png]

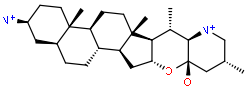

Supplement: Supplementary file 1 [file Data_Sheet_1.ZIP › supplementary/ligands-with-tautomers/structures/mol-200.png]

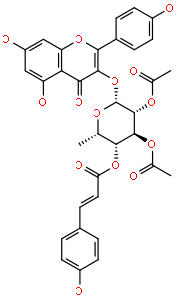

Supplement: Supplementary file 1 [file Data_Sheet_1.ZIP › supplementary/ligands-with-tautomers/structures/mol-214.png]

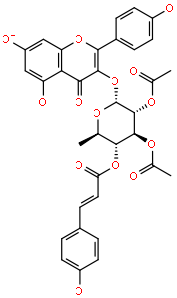

Supplement: Supplementary file 1 [file Data_Sheet_1.ZIP › supplementary/ligands-with-tautomers/structures/mol-238.png]

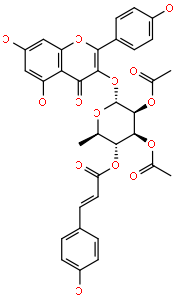

Supplement: Supplementary file 1 [file Data_Sheet_1.ZIP › supplementary/ligands-with-tautomers/structures/mol-210.png]

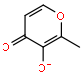

Supplement: Supplementary file 1 [file Data_Sheet_1.ZIP › supplementary/ligands-with-tautomers/structures/mol-204.png]

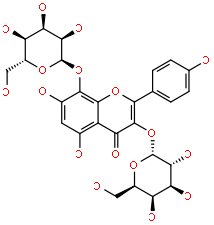

Supplement: Supplementary file 1 [file Data_Sheet_1.ZIP › supplementary/ligands-with-tautomers/structures/mol-012.png]

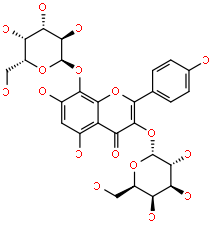

Supplement: Supplementary file 1 [file Data_Sheet_1.ZIP › supplementary/ligands-with-tautomers/structures/mol-006.png]

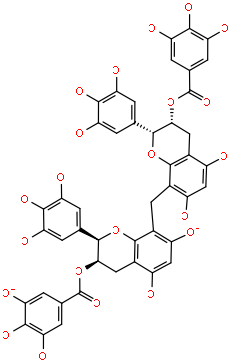

Supplement: Supplementary file 1 [file Data_Sheet_1.ZIP › supplementary/ligands-with-tautomers/structures/mol-166.png]

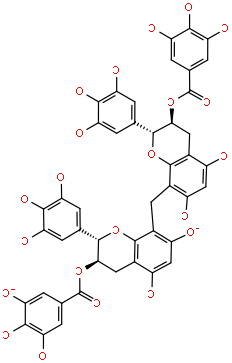

Supplement: Supplementary file 1 [file Data_Sheet_1.ZIP › supplementary/ligands-with-tautomers/structures/mol-172.png]

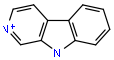

Supplement: Supplementary file 1 [file Data_Sheet_1.ZIP › supplementary/ligands-with-tautomers/structures/mol-198.png]

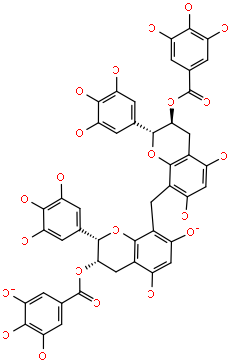

Supplement: Supplementary file 1 [file Data_Sheet_1.ZIP › supplementary/ligands-with-tautomers/structures/mol-173.png]

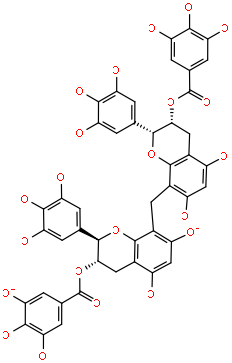

Supplement: Supplementary file 1 [file Data_Sheet_1.ZIP › supplementary/ligands-with-tautomers/structures/mol-167.png]

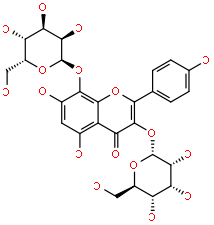

Supplement: Supplementary file 1 [file Data_Sheet_1.ZIP › supplementary/ligands-with-tautomers/structures/mol-007.png]

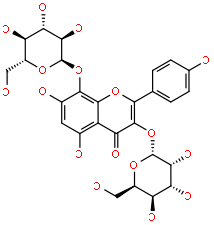

Supplement: Supplementary file 1 [file Data_Sheet_1.ZIP › supplementary/ligands-with-tautomers/structures/mol-013.png]

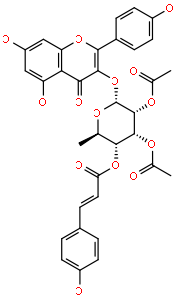

Supplement: Supplementary file 1 [file Data_Sheet_1.ZIP › supplementary/ligands-with-tautomers/structures/mol-205.png]

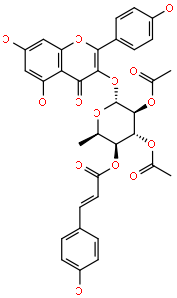

Supplement: Supplementary file 1 [file Data_Sheet_1.ZIP › supplementary/ligands-with-tautomers/structures/mol-211.png]

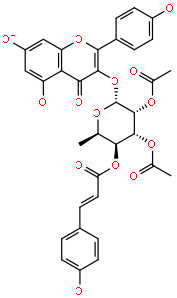

Supplement: Supplementary file 1 [file Data_Sheet_1.ZIP › supplementary/ligands-with-tautomers/structures/mol-239.png]

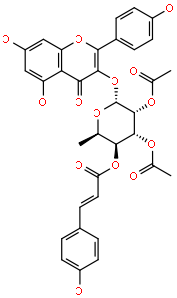

Supplement: Supplementary file 1 [file Data_Sheet_1.ZIP › supplementary/ligands-with-tautomers/structures/mol-207.png]

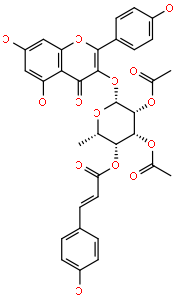

Supplement: Supplementary file 1 [file Data_Sheet_1.ZIP › supplementary/ligands-with-tautomers/structures/mol-213.png]

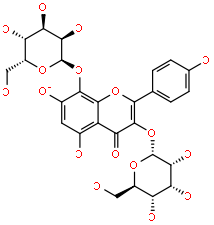

Supplement: Supplementary file 1 [file Data_Sheet_1.ZIP › supplementary/ligands-with-tautomers/structures/mol-039.png]

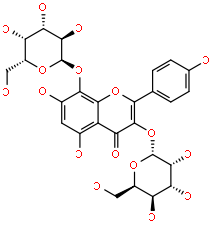

Supplement: Supplementary file 1 [file Data_Sheet_1.ZIP › supplementary/ligands-with-tautomers/structures/mol-005.png]

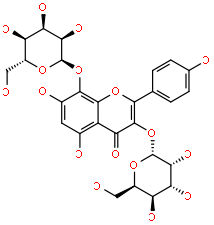

Supplement: Supplementary file 1 [file Data_Sheet_1.ZIP › supplementary/ligands-with-tautomers/structures/mol-011.png]

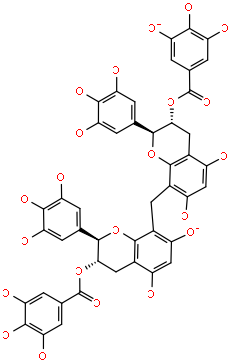

Supplement: Supplementary file 1 [file Data_Sheet_1.ZIP › supplementary/ligands-with-tautomers/structures/mol-159.png]

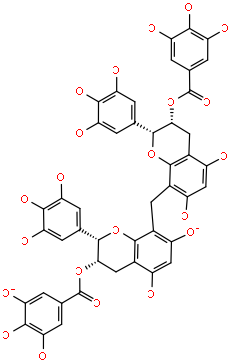

Supplement: Supplementary file 1 [file Data_Sheet_1.ZIP › supplementary/ligands-with-tautomers/structures/mol-171.png]

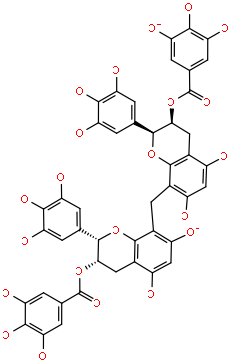

Supplement: Supplementary file 1 [file Data_Sheet_1.ZIP › supplementary/ligands-with-tautomers/structures/mol-165.png]

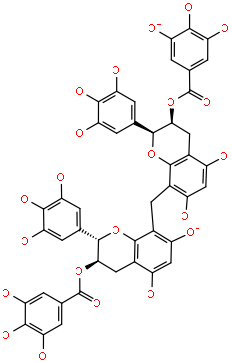

Supplement: Supplementary file 1 [file Data_Sheet_1.ZIP › supplementary/ligands-with-tautomers/structures/mol-164.png]

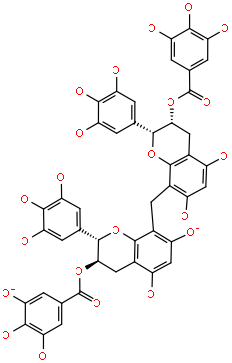

Supplement: Supplementary file 1 [file Data_Sheet_1.ZIP › supplementary/ligands-with-tautomers/structures/mol-170.png]

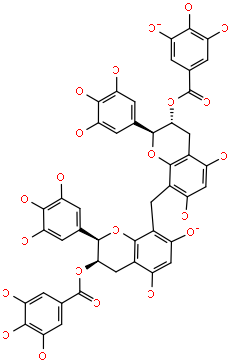

Supplement: Supplementary file 1 [file Data_Sheet_1.ZIP › supplementary/ligands-with-tautomers/structures/mol-158.png]

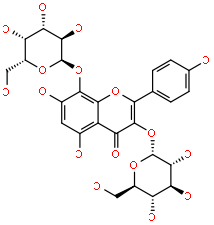

Supplement: Supplementary file 1 [file Data_Sheet_1.ZIP › supplementary/ligands-with-tautomers/structures/mol-010.png]

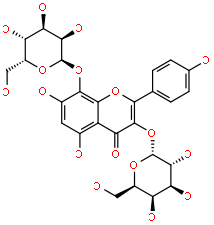

Supplement: Supplementary file 1 [file Data_Sheet_1.ZIP › supplementary/ligands-with-tautomers/structures/mol-004.png]

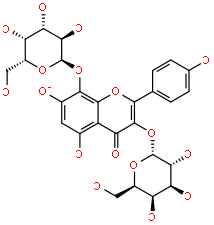

Supplement: Supplementary file 1 [file Data_Sheet_1.ZIP › supplementary/ligands-with-tautomers/structures/mol-038.png]

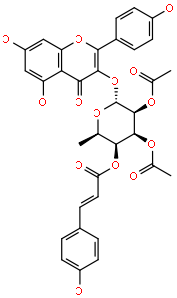

Supplement: Supplementary file 1 [file Data_Sheet_1.ZIP › supplementary/ligands-with-tautomers/structures/mol-212.png]

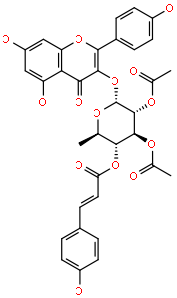

Supplement: Supplementary file 1 [file Data_Sheet_1.ZIP › supplementary/ligands-with-tautomers/structures/mol-206.png]

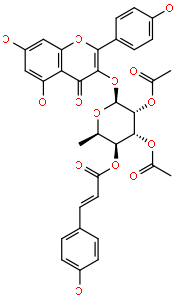

Supplement: Supplementary file 1 [file Data_Sheet_1.ZIP › supplementary/ligands-with-tautomers/structures/mol-223.png]

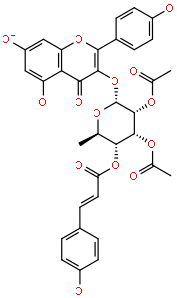

Supplement: Supplementary file 1 [file Data_Sheet_1.ZIP › supplementary/ligands-with-tautomers/structures/mol-237.png]

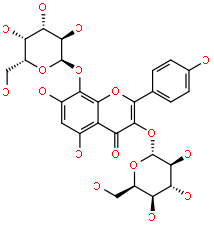

Supplement: Supplementary file 1 [file Data_Sheet_1.ZIP › supplementary/ligands-with-tautomers/structures/mol-021.png]

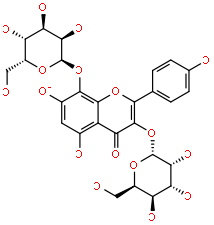

Supplement: Supplementary file 1 [file Data_Sheet_1.ZIP › supplementary/ligands-with-tautomers/structures/mol-035.png]

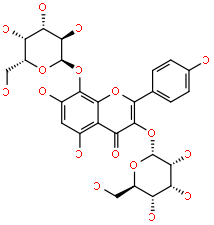

Supplement: Supplementary file 1 [file Data_Sheet_1.ZIP › supplementary/ligands-with-tautomers/structures/mol-009.png]

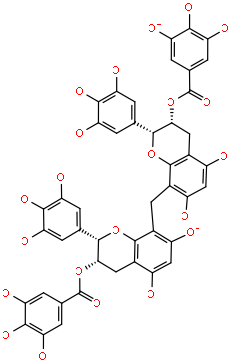

Supplement: Supplementary file 1 [file Data_Sheet_1.ZIP › supplementary/ligands-with-tautomers/structures/mol-155.png]
