# Supplementary figures and images for: Chemical and biochemical characterization of Ipomoea aquatica: genoprotective potential and inhibitory mechanism of its phytochemicals against α-amylase and α-glucosidase (part 1 of 2)
Source: Front Nutr. 2023 Dec 21;10:1304903. doi: 10.3389/fnut.2023.1304903 (PMC10772144; doi:10.3389/fnut.2023.1304903)

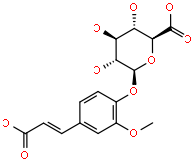

Supplement: Supplementary file 1 [file Data_Sheet_1.ZIP › supplementary/ligands/structures/mol-014.png]

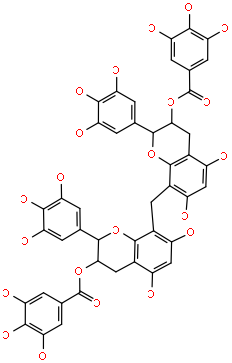

Supplement: Supplementary file 1 [file Data_Sheet_1.ZIP › supplementary/ligands/structures/mol-028.png]

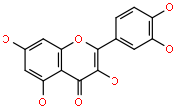

Supplement: Supplementary file 1 [file Data_Sheet_1.ZIP › supplementary/ligands/structures/mol-029.png]

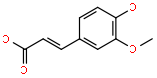

Supplement: Supplementary file 1 [file Data_Sheet_1.ZIP › supplementary/ligands/structures/mol-015.png]

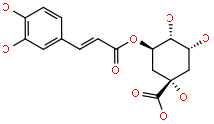

Supplement: Supplementary file 1 [file Data_Sheet_1.ZIP › supplementary/ligands/structures/mol-001.png]

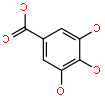

Supplement: Supplementary file 1 [file Data_Sheet_1.ZIP › supplementary/ligands/structures/mol-017.png]

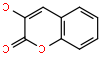

Supplement: Supplementary file 1 [file Data_Sheet_1.ZIP › supplementary/ligands/structures/mol-003.png]

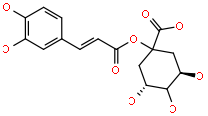

Supplement: Supplementary file 1 [file Data_Sheet_1.ZIP › supplementary/ligands/structures/mol-002.png]

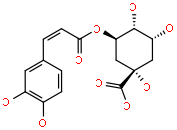

Supplement: Supplementary file 1 [file Data_Sheet_1.ZIP › supplementary/ligands/structures/mol-016.png]

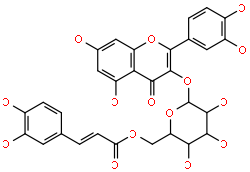

Supplement: Supplementary file 1 [file Data_Sheet_1.ZIP › supplementary/ligands/structures/mol-006.png]

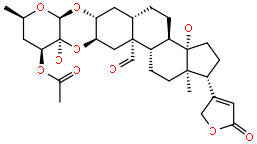

Supplement: Supplementary file 1 [file Data_Sheet_1.ZIP › supplementary/ligands/structures/mol-007.png]

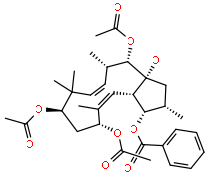

Supplement: Supplementary file 1 [file Data_Sheet_1.ZIP › supplementary/ligands/structures/mol-013.png]

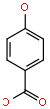

Supplement: Supplementary file 1 [file Data_Sheet_1.ZIP › supplementary/ligands/structures/mol-005.png]

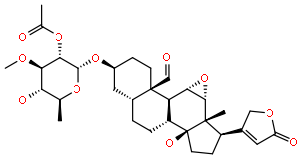

Supplement: Supplementary file 1 [file Data_Sheet_1.ZIP › supplementary/ligands/structures/mol-010.png]

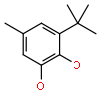

Supplement: Supplementary file 1 [file Data_Sheet_1.ZIP › supplementary/ligands/structures/mol-004.png]

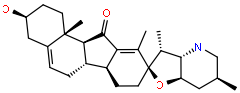

Supplement: Supplementary file 1 [file Data_Sheet_1.ZIP › supplementary/ligands/structures/mol-021.png]

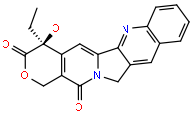

Supplement: Supplementary file 1 [file Data_Sheet_1.ZIP › supplementary/ligands/structures/mol-009.png]

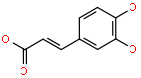

Supplement: Supplementary file 1 [file Data_Sheet_1.ZIP › supplementary/ligands/structures/mol-008.png]

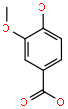

Supplement: Supplementary file 1 [file Data_Sheet_1.ZIP › supplementary/ligands/structures/mol-034.png]

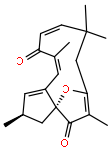

Supplement: Supplementary file 1 [file Data_Sheet_1.ZIP › supplementary/ligands/structures/mol-020.png]

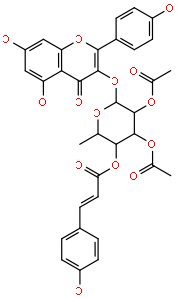

Supplement: Supplementary file 1 [file Data_Sheet_1.ZIP › supplementary/ligands/structures/mol-022.png]

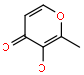

Supplement: Supplementary file 1 [file Data_Sheet_1.ZIP › supplementary/ligands/structures/mol-023.png]

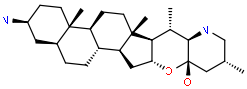

Supplement: Supplementary file 1 [file Data_Sheet_1.ZIP › supplementary/ligands/structures/mol-033.png]

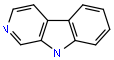

Supplement: Supplementary file 1 [file Data_Sheet_1.ZIP › supplementary/ligands/structures/mol-027.png]

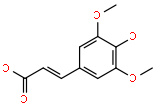

Supplement: Supplementary file 1 [file Data_Sheet_1.ZIP › supplementary/ligands/structures/mol-032.png]

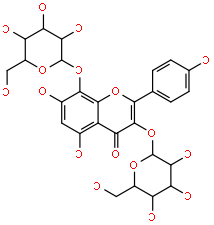

Supplement: Supplementary file 1 [file Data_Sheet_1.ZIP › supplementary/ligands/structures/mol-018.png]

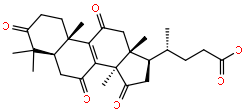

Supplement: Supplementary file 1 [file Data_Sheet_1.ZIP › supplementary/ligands/structures/mol-024.png]

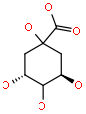

Supplement: Supplementary file 1 [file Data_Sheet_1.ZIP › supplementary/ligands/structures/mol-030.png]

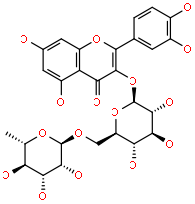

Supplement: Supplementary file 1 [file Data_Sheet_1.ZIP › supplementary/ligands/structures/mol-031.png]

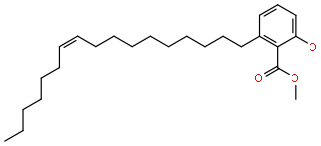

Supplement: Supplementary file 1 [file Data_Sheet_1.ZIP › supplementary/ligands/structures/mol-025.png]

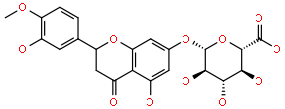

Supplement: Supplementary file 1 [file Data_Sheet_1.ZIP › supplementary/ligands/structures/mol-019.png]

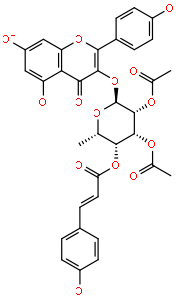

Supplement: Supplementary file 1 [file Data_Sheet_1.ZIP › supplementary/ligands-with-tautomers/structures/mol-261.png]

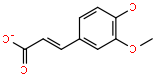

Supplement: Supplementary file 1 [file Data_Sheet_1.ZIP › supplementary/ligands-with-tautomers/structures/mol-275.png]

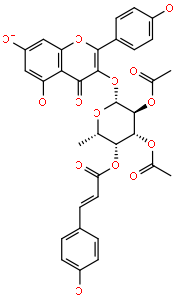

Supplement: Supplementary file 1 [file Data_Sheet_1.ZIP › supplementary/ligands-with-tautomers/structures/mol-249.png]

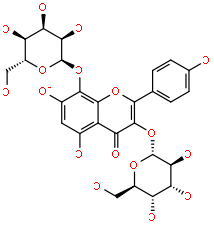

Supplement: Supplementary file 1 [file Data_Sheet_1.ZIP › supplementary/ligands-with-tautomers/structures/mol-063.png]

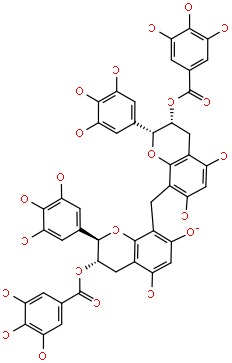

Supplement: Supplementary file 1 [file Data_Sheet_1.ZIP › supplementary/ligands-with-tautomers/structures/mol-077.png]

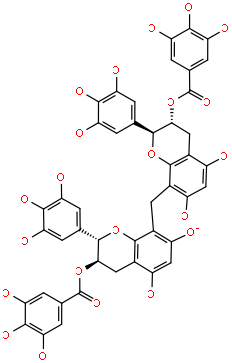

Supplement: Supplementary file 1 [file Data_Sheet_1.ZIP › supplementary/ligands-with-tautomers/structures/mol-088.png]

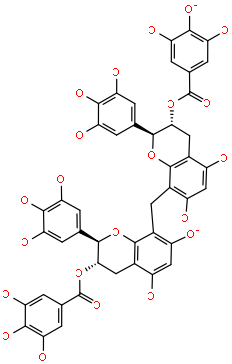

Supplement: Supplementary file 1 [file Data_Sheet_1.ZIP › supplementary/ligands-with-tautomers/structures/mol-117.png]

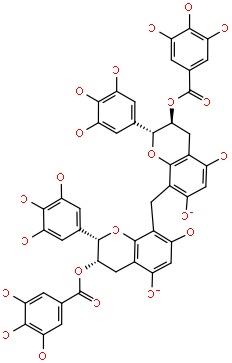

Supplement: Supplementary file 1 [file Data_Sheet_1.ZIP › supplementary/ligands-with-tautomers/structures/mol-103.png]

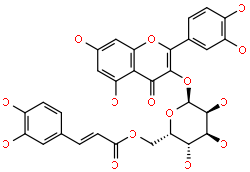

Supplement: Supplementary file 1 [file Data_Sheet_1.ZIP › supplementary/ligands-with-tautomers/structures/mol-315.png]

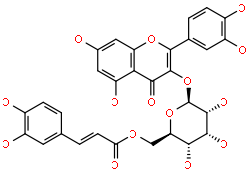

Supplement: Supplementary file 1 [file Data_Sheet_1.ZIP › supplementary/ligands-with-tautomers/structures/mol-301.png]

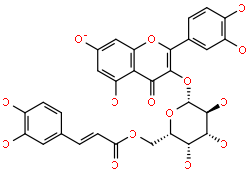

Supplement: Supplementary file 1 [file Data_Sheet_1.ZIP › supplementary/ligands-with-tautomers/structures/mol-329.png]

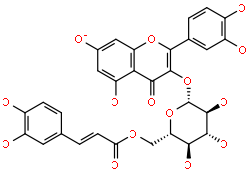

Supplement: Supplementary file 1 [file Data_Sheet_1.ZIP › supplementary/ligands-with-tautomers/structures/mol-328.png]

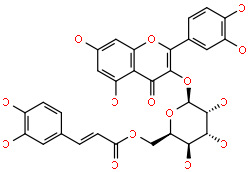

Supplement: Supplementary file 1 [file Data_Sheet_1.ZIP › supplementary/ligands-with-tautomers/structures/mol-300.png]

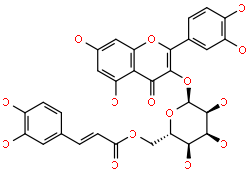

Supplement: Supplementary file 1 [file Data_Sheet_1.ZIP › supplementary/ligands-with-tautomers/structures/mol-314.png]

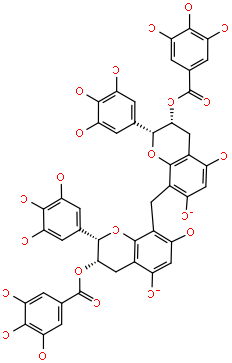

Supplement: Supplementary file 1 [file Data_Sheet_1.ZIP › supplementary/ligands-with-tautomers/structures/mol-102.png]

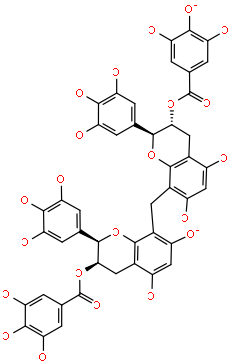

Supplement: Supplementary file 1 [file Data_Sheet_1.ZIP › supplementary/ligands-with-tautomers/structures/mol-116.png]

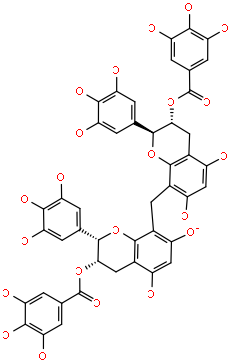

Supplement: Supplementary file 1 [file Data_Sheet_1.ZIP › supplementary/ligands-with-tautomers/structures/mol-089.png]

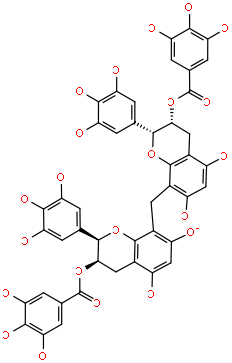

Supplement: Supplementary file 1 [file Data_Sheet_1.ZIP › supplementary/ligands-with-tautomers/structures/mol-076.png]

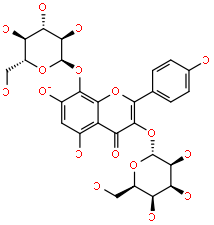

Supplement: Supplementary file 1 [file Data_Sheet_1.ZIP › supplementary/ligands-with-tautomers/structures/mol-062.png]

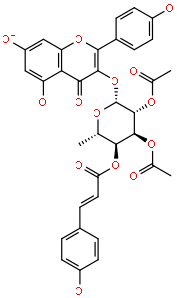

Supplement: Supplementary file 1 [file Data_Sheet_1.ZIP › supplementary/ligands-with-tautomers/structures/mol-248.png]

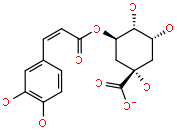

Supplement: Supplementary file 1 [file Data_Sheet_1.ZIP › supplementary/ligands-with-tautomers/structures/mol-274.png]

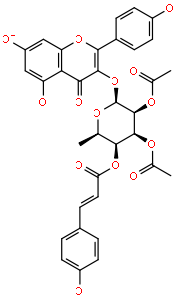

Supplement: Supplementary file 1 [file Data_Sheet_1.ZIP › supplementary/ligands-with-tautomers/structures/mol-260.png]

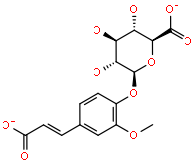

Supplement: Supplementary file 1 [file Data_Sheet_1.ZIP › supplementary/ligands-with-tautomers/structures/mol-276.png]

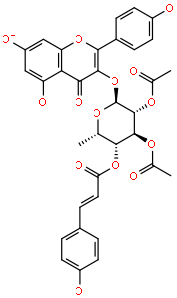

Supplement: Supplementary file 1 [file Data_Sheet_1.ZIP › supplementary/ligands-with-tautomers/structures/mol-262.png]

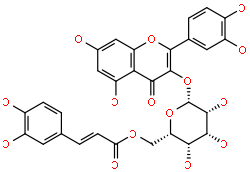

Supplement: Supplementary file 1 [file Data_Sheet_1.ZIP › supplementary/ligands-with-tautomers/structures/mol-289.png]

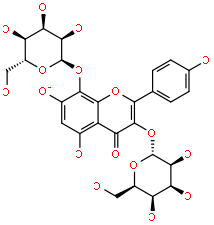

Supplement: Supplementary file 1 [file Data_Sheet_1.ZIP › supplementary/ligands-with-tautomers/structures/mol-060.png]

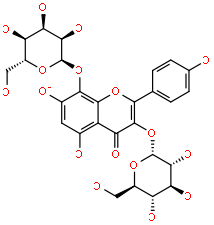

Supplement: Supplementary file 1 [file Data_Sheet_1.ZIP › supplementary/ligands-with-tautomers/structures/mol-048.png]

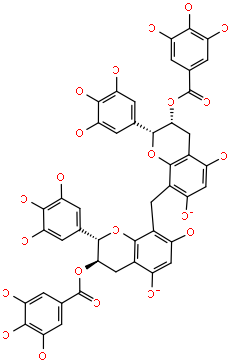

Supplement: Supplementary file 1 [file Data_Sheet_1.ZIP › supplementary/ligands-with-tautomers/structures/mol-100.png]

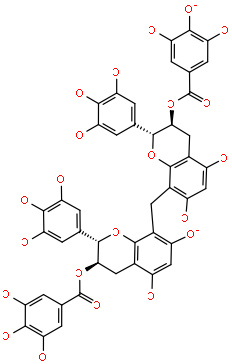

Supplement: Supplementary file 1 [file Data_Sheet_1.ZIP › supplementary/ligands-with-tautomers/structures/mol-114.png]

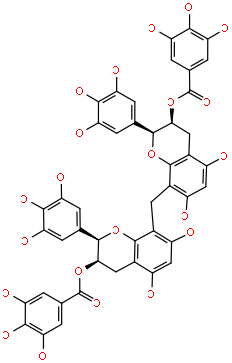

Supplement: Supplementary file 1 [file Data_Sheet_1.ZIP › supplementary/ligands-with-tautomers/structures/mol-128.png]

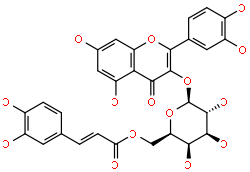

Supplement: Supplementary file 1 [file Data_Sheet_1.ZIP › supplementary/ligands-with-tautomers/structures/mol-302.png]

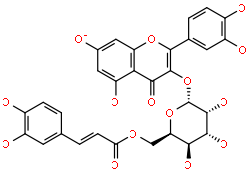

Supplement: Supplementary file 1 [file Data_Sheet_1.ZIP › supplementary/ligands-with-tautomers/structures/mol-316.png]

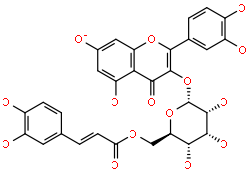

Supplement: Supplementary file 1 [file Data_Sheet_1.ZIP › supplementary/ligands-with-tautomers/structures/mol-317.png]

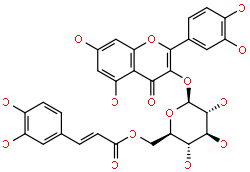

Supplement: Supplementary file 1 [file Data_Sheet_1.ZIP › supplementary/ligands-with-tautomers/structures/mol-303.png]

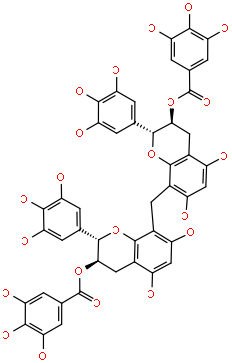

Supplement: Supplementary file 1 [file Data_Sheet_1.ZIP › supplementary/ligands-with-tautomers/structures/mol-129.png]

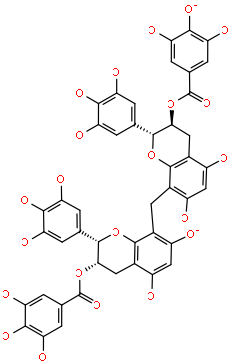

Supplement: Supplementary file 1 [file Data_Sheet_1.ZIP › supplementary/ligands-with-tautomers/structures/mol-115.png]

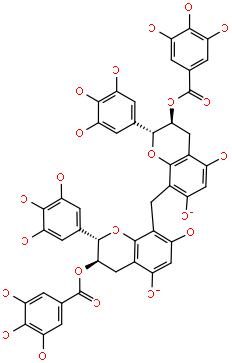

Supplement: Supplementary file 1 [file Data_Sheet_1.ZIP › supplementary/ligands-with-tautomers/structures/mol-101.png]

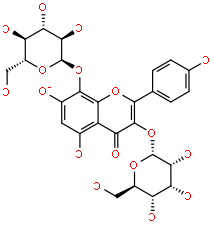

Supplement: Supplementary file 1 [file Data_Sheet_1.ZIP › supplementary/ligands-with-tautomers/structures/mol-049.png]

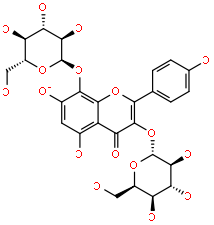

Supplement: Supplementary file 1 [file Data_Sheet_1.ZIP › supplementary/ligands-with-tautomers/structures/mol-061.png]

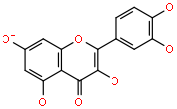

Supplement: Supplementary file 1 [file Data_Sheet_1.ZIP › supplementary/ligands-with-tautomers/structures/mol-075.png]

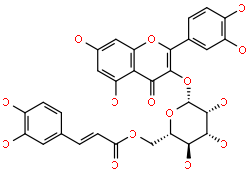

Supplement: Supplementary file 1 [file Data_Sheet_1.ZIP › supplementary/ligands-with-tautomers/structures/mol-288.png]

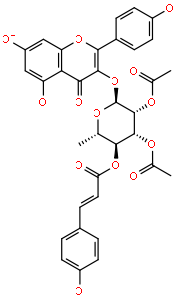

Supplement: Supplementary file 1 [file Data_Sheet_1.ZIP › supplementary/ligands-with-tautomers/structures/mol-263.png]

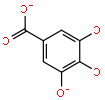

Supplement: Supplementary file 1 [file Data_Sheet_1.ZIP › supplementary/ligands-with-tautomers/structures/mol-273.png]

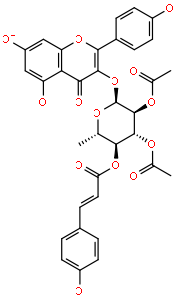

Supplement: Supplementary file 1 [file Data_Sheet_1.ZIP › supplementary/ligands-with-tautomers/structures/mol-267.png]

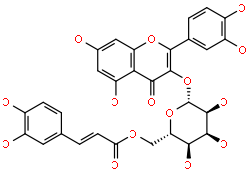

Supplement: Supplementary file 1 [file Data_Sheet_1.ZIP › supplementary/ligands-with-tautomers/structures/mol-298.png]

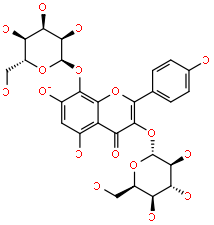

Supplement: Supplementary file 1 [file Data_Sheet_1.ZIP › supplementary/ligands-with-tautomers/structures/mol-059.png]

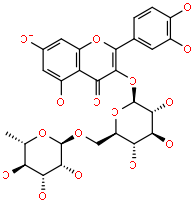

Supplement: Supplementary file 1 [file Data_Sheet_1.ZIP › supplementary/ligands-with-tautomers/structures/mol-071.png]

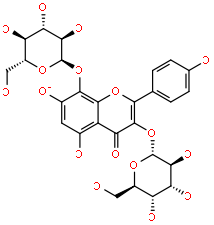

Supplement: Supplementary file 1 [file Data_Sheet_1.ZIP › supplementary/ligands-with-tautomers/structures/mol-065.png]

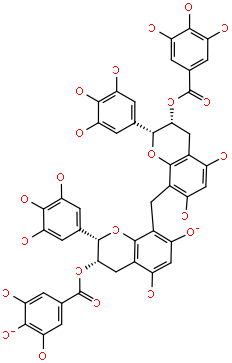

Supplement: Supplementary file 1 [file Data_Sheet_1.ZIP › supplementary/ligands-with-tautomers/structures/mol-139.png]

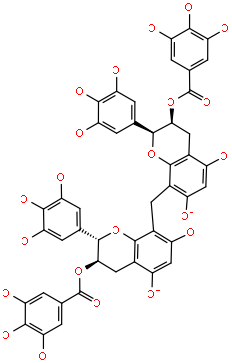

Supplement: Supplementary file 1 [file Data_Sheet_1.ZIP › supplementary/ligands-with-tautomers/structures/mol-105.png]

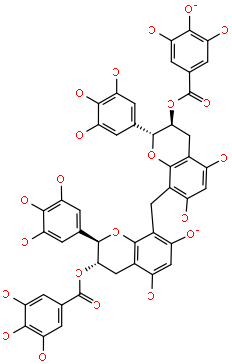

Supplement: Supplementary file 1 [file Data_Sheet_1.ZIP › supplementary/ligands-with-tautomers/structures/mol-111.png]

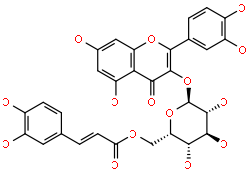

Supplement: Supplementary file 1 [file Data_Sheet_1.ZIP › supplementary/ligands-with-tautomers/structures/mol-307.png]

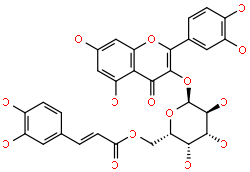

Supplement: Supplementary file 1 [file Data_Sheet_1.ZIP › supplementary/ligands-with-tautomers/structures/mol-313.png]

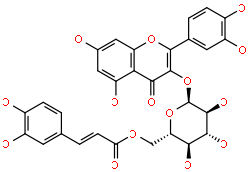

Supplement: Supplementary file 1 [file Data_Sheet_1.ZIP › supplementary/ligands-with-tautomers/structures/mol-312.png]

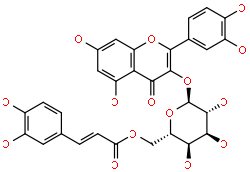

Supplement: Supplementary file 1 [file Data_Sheet_1.ZIP › supplementary/ligands-with-tautomers/structures/mol-306.png]

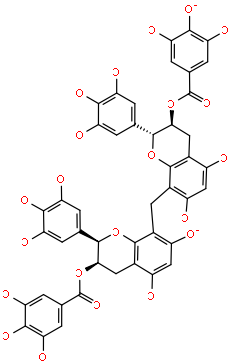

Supplement: Supplementary file 1 [file Data_Sheet_1.ZIP › supplementary/ligands-with-tautomers/structures/mol-110.png]

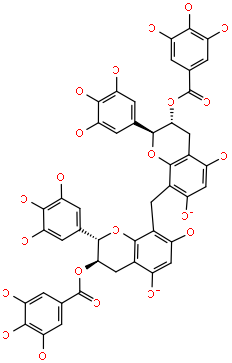

Supplement: Supplementary file 1 [file Data_Sheet_1.ZIP › supplementary/ligands-with-tautomers/structures/mol-104.png]

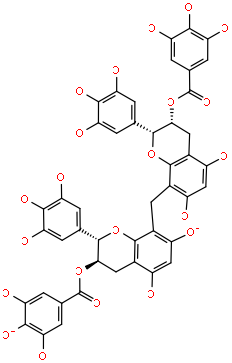

Supplement: Supplementary file 1 [file Data_Sheet_1.ZIP › supplementary/ligands-with-tautomers/structures/mol-138.png]

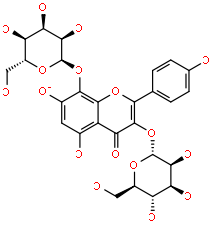

Supplement: Supplementary file 1 [file Data_Sheet_1.ZIP › supplementary/ligands-with-tautomers/structures/mol-064.png]

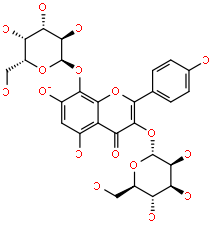

Supplement: Supplementary file 1 [file Data_Sheet_1.ZIP › supplementary/ligands-with-tautomers/structures/mol-058.png]

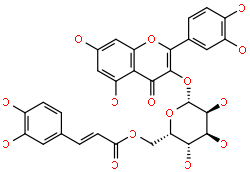

Supplement: Supplementary file 1 [file Data_Sheet_1.ZIP › supplementary/ligands-with-tautomers/structures/mol-299.png]

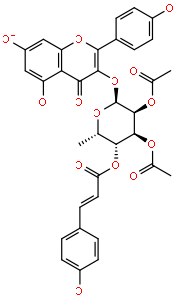

Supplement: Supplementary file 1 [file Data_Sheet_1.ZIP › supplementary/ligands-with-tautomers/structures/mol-266.png]

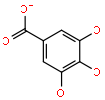

Supplement: Supplementary file 1 [file Data_Sheet_1.ZIP › supplementary/ligands-with-tautomers/structures/mol-272.png]

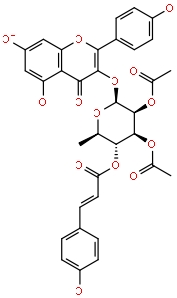

Supplement: Supplementary file 1 [file Data_Sheet_1.ZIP › supplementary/ligands-with-tautomers/structures/mol-258.png]

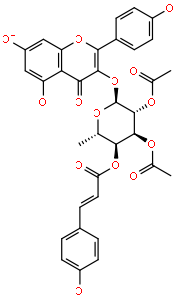

Supplement: Supplementary file 1 [file Data_Sheet_1.ZIP › supplementary/ligands-with-tautomers/structures/mol-264.png]

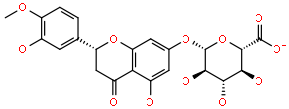

Supplement: Supplementary file 1 [file Data_Sheet_1.ZIP › supplementary/ligands-with-tautomers/structures/mol-270.png]

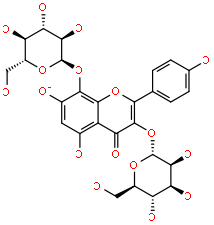

Supplement: Supplementary file 1 [file Data_Sheet_1.ZIP › supplementary/ligands-with-tautomers/structures/mol-066.png]

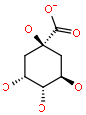

Supplement: Supplementary file 1 [file Data_Sheet_1.ZIP › supplementary/ligands-with-tautomers/structures/mol-072.png]

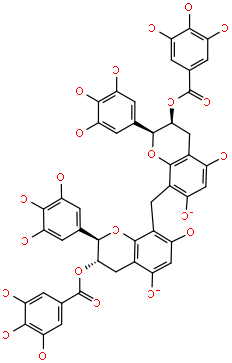

Supplement: Supplementary file 1 [file Data_Sheet_1.ZIP › supplementary/ligands-with-tautomers/structures/mol-099.png]
